# Global proteogenomic analysis of human MHC class I-associated peptides derived from non-canonical reading frames

**DOI:** 10.1038/ncomms10238

**Published:** 2016-01-05

**Authors:** Céline M. Laumont, Tariq Daouda, Jean-Philippe Laverdure, Éric Bonneil, Olivier Caron-Lizotte, Marie-Pierre Hardy, Diana P. Granados, Chantal Durette, Sébastien Lemieux, Pierre Thibault, Claude Perreault

**Affiliations:** 1 Institute for Research in Immunology and Cancer, Université de Montréal, PO Box 6128 Station Centre-Ville, Montreal, Quebec, Canada H3C 3J7; 2 Department of Medicine, Faculty of Medicine, Université de Montréal, PO Box 6128 Station Centre-Ville, Montreal, Quebec, Canada H3C 3J7; 3 Department of Computer Science and Operations Research, Faculty of Arts and Sciences, Université de Montréal, PO Box 6128 Station Centre-Ville, Montreal, Quebec, Canada H3C 3J7; 4 Department of Chemistry, Université de Montréal, PO Box 6128 Station Centre-Ville, Montreal, Quebec, Canada H3C 3J7; 5 Division of Hematology, Hôpital Maisonneuve-Rosemont, 5415 de l'Assomption Boulevard, Montreal, Quebec, Canada H1T 2M4

## Abstract

In view of recent reports documenting pervasive translation outside of canonical protein-coding sequences, we wished to determine the proportion of major histocompatibility complex (MHC) class I-associated peptides (MAPs) derived from non-canonical reading frames. Here we perform proteogenomic analyses of MAPs eluted from human B cells using high-throughput mass spectrometry to probe the six-frame translation of the B-cell transcriptome. We report that ∼10% of MAPs originate from allegedly noncoding genomic sequences or exonic out-of-frame translation. The biogenesis and properties of these ‘cryptic MAPs' differ from those of conventional MAPs. Cryptic MAPs come from very short proteins with atypical C termini, and are coded by transcripts bearing long 3′UTRs enriched in destabilizing elements. Relative to conventional MAPs, cryptic MAPs display different MHC class I-binding preferences and harbour more genomic polymorphisms, some of which are immunogenic. Cryptic MAPs increase the complexity of the MAP repertoire and enhance the scope of CD8 T-cell immunosurveillance.

## Abstract (web summary)

Cryptic translation of the 'non-coding' genome is increasingly recognised, however its biological significance remains unclear. Laumont *et al.* employ proteogenomic techniques to map the human immunoproteome, and find that approximately 10% of MHC class I-associated peptides are cryptic.

## Graphical abstract

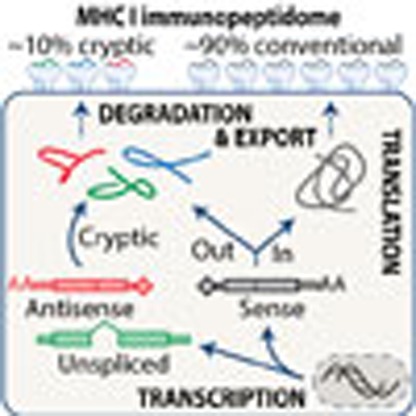

Breathtaking advances in genomics and proteomics are drastically changing our perspective of cell biology and, in particular, our understanding of protein synthesis and degradation. For instance, next-generation sequencing analyses have shown that three-quarters of the human genome is capable of being transcribed^1^. Meanwhile, high-throughput mass spectrometry (MS) studies in normal and infected human cells have resulted in the identification of proteins representing more than 80% of canonical human and viral protein-coding genes^2^,^3^. Recently, a quantum leap in systems biology was made possible by the emergence of a new field, proteogenomics, that leverages on next-generation sequencing to perform ‘genomically informed proteomics'^4^. In conventional shotgun proteomics, peptide sequencing is achieved by matching tandem MS spectra from an experimental sample against a reference protein sequence database (for example, UniProt). As a result, conventional MS sequencing suffers from a major limitation: it can only identify peptides encoded by the canonical reading frame of classic exons. The crux of proteogenomic studies is to perform MS-based peptide sequencing by searching customized databases containing the six-frame translation of genomic or transcriptomic sequences. In this way, proteogenomics studies can identify peptides encoded by all reading frames of any genomic region^5^.

Proteogenomics has rapidly revolutionized our vision of the proteome of cells from numerous living organisms, including normal and neoplastic human cells^4^,^5^. A fundamental issue tackled by proteogenomics is the landscape of genomic regions that are expressed at the protein level. Ribosome-profiling experiments have provided strong evidence for pervasive translation outside of annotated protein-coding genes^6^. However, the definite proof of a genomic locus being protein-coding is the detection of its corresponding protein^7^. Accordingly, one salient concept emerging from proteogenomic analyses is that the proteome is more complex than previously thought. The proteome contains peptides arising from a variety of RNAs that were not supposed to encode proteins (noncoding RNAs) and are therefore not included in annotated protein databases. Many long noncoding RNAs, short open reading frames (ORFs) and pseudogenes, mislabelled as ‘noncoding', were ultimately found to code for peptides^2^,^3^,^4^,^5^,^6^,^7^,^8^,^9^. Moreover, numerous peptides originate from non-canonical reading frames with non-AUG start codons^10^.

We therefore hypothesised that proteogenomics might allow us to elucidate a fundamental question: the contribution of proteins derived from non-canonical transcripts to the repertoire of major histocompatibility complex (MHC) class I-associated peptides (MAPs). Endogenous MAPs are collectively referred to as the immunopeptidome and represent the essence of self for CD8 T lymphocytes^11^,^12^. Despite the fundamental importance of the immunopeptidome, its genesis remains ill-defined^13^,^14^. MAPs derive from proteolytic degradation of proteins found in all cell compartments; however, the immunopeptidome is not a random sample of the proteome: many abundant proteins do not generate MAPs, while some low-abundance proteins generate large amounts of MAPs^13^,^14^,^15^,^16^. In a series of seminal studies, Shastri and colleagues made startling observations showing that, similarly to the proteome, the immunopeptidome might be more complex than anticipated. Using an alloreactive T-cell clone as a probe, they screened a splenic cDNA library in transfected antigen-presenting cells (APCs) and isolated a cDNA clone that encoded the MAP recognized by the T-cell clone. The salient finding was that this MAP derived from a non-canonical reading frame initiated with a non-AUG start codon^17^. They discovered that synthesis of this peptide was initiated with a CUG codon decoded as a leucine rather than a methionine^18^. Studies by other groups provided evidence that MAPs could arise not only from alternate translational reading frames but also from untranslated regions (UTRs or introns)^19^,^20^. However, the structure of only a handful of these ‘cryptic MAPs' has been confirmed with MS^20^,^21^. Therefore, in the absence of proteomic evidence, the existence of most reported cryptic MAPs must be considered with some scepticism because their identification relied on indirect methods fraught with high false discovery rates (FDRs). We therefore developed a novel proteogenomic approach to define the landscape of the cryptic immunopeptidome and answer the following questions: what proportion of MAPs derives from non-canonical reading frames and how are they generated? To this end, we performed an all-frames translation of the transcriptome of human B lymphoblastoid cell lines to generate databases of predicted peptides/proteins. These databases were used to identify MAPs using high-throughput MS sequencing. Integration of transcriptomic and proteomic data revealed that cryptic MAPs constitute ∼10% of the immunopeptidome and that their biogenesis and properties differ in many ways from those of conventional MAPs.

## Results

### Novel proteogenomic strategy to identify cryptic MAPs

**Figure 1:**
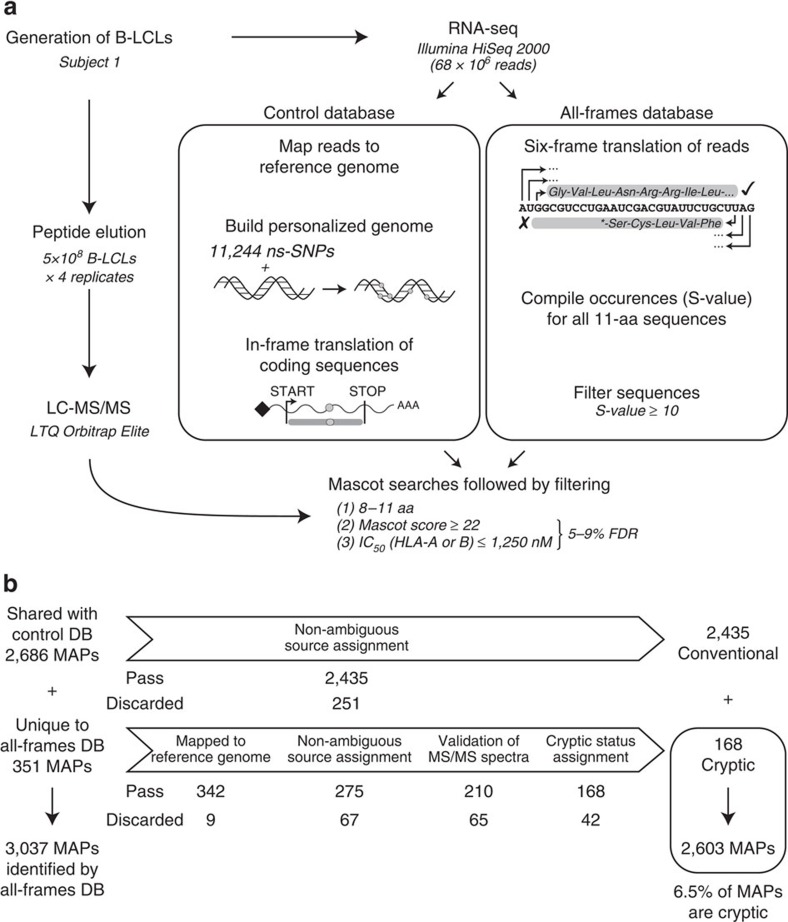
Proteogenomic workflow used for high-throughput identification of cryptic MAPs. (**a**) General overview of the proteogenomic workflow used to identify conventional (Conv.) and cryptic (Crypt.) MAPs. Peptides were eluted from the cell surface of subject 1's B-LCL and were sequenced with liquid chromatography-MS/MS (LC-MS/MS). To determine the amino-acid (aa) sequence of those peptides, we built two databases (DBs), both derived from the analysis of RNA-seq data obtained from subject 1's B-LCL: the control DB and the all-frames DB (see Methods and Supplementary Fig. 1). (**b**) Peptides solely identified by the all-frames DB were considered as Crypt. MAP candidates and further filtered to remove ambiguous and false-positive identifications. See also Supplementary Figs 2 and 3.

MAPs were eluted from an Epstein-Barr virus-transformed B-cell line (B-LCL) obtained from a blood donor bearing the HLA-A*03:01, -A*29:02; -B*08:01, -B*44:03 MHC class I molecules (referred to as subject 1). Peptides were fractionated with strong cation exchange chromatography and analysed with liquid chromatography-MS/MS using high-resolution precursor and product ion spectra, as previously described^22^. To identify both conventional and cryptic MAPs present at the surface of this B-LCL, peptides were matched to two personalized databases referred to as the ‘control' and the ‘all-frames' databases (Fig. 1a). Both databases were built by *in silico* translation of RNA-sequencing (RNA-seq) data from subject 1's B-LCL using the pyGeno python package (https://github.com/tariqdaouda/pyGeno)^23^. Two reasons led us to focus on the transcriptome rather than the genome of our B-LCL for database construction: (i) MAPs can only derive from transcripts expressed in the cell of interest and (ii) in proteogenomics, the risk of false discovery increases with the size of the database used for MS sequencing^4^,^5^.

The control database corresponds to the canonical proteome of the B-LCL and was generated as follows (Fig. 1a, left): RNA-seq reads were mapped on the reference genome (version GRCh37.75) to identify subject 1-specific high-quality non-synonymous single-nucleotide polymorphisms (ns-SNPs), which were then integrated in the reference genome to build the personalized genome of subject 1. All putative protein-coding genes were then *in silico* translated in their conventional reading frame to obtain the canonical proteome of the B-LCL. The all-frames database was built using the six-frame translation of RNA-seq data from the B-LCL (Fig. 1a, right): reads passing the Illumina quality filters were *in silico* translated into six possible reading frames using a sliding window of 33 base pairs (bp), since the vast majority of MAPs are known to be 8–11 amino acids long and only rare MAPs contain more than 11 residues. Translation products having a length inferior to eight amino acids, due to the presence of a stop codon within the sliding window, were excluded. By not aligning the reads before translating them, we are able to leverage the whole output of the sequencer, including reads resulting from rare elongation events that might otherwise be discarded. However, this approach also prevents us from using established filtering approaches such as coverage measures or base quality filters. To address the necessity of sequence filtering, we computed for each translated peptide an *S*-value (or Seen-value) that represents the number of times a peptide was seen following the *in silico* translation (Supplementary Fig. 1a). The higher the *S*-value, the more confidence we have that the peptide sequence is indeed not due to a sequencing error. We therefore elected to use a stringent approach and kept only peptides having an *S*-value≥10 to (i) obtain a database whose size was manageable using the Mascot search engine (Supplementary Fig. 1b) and (ii) to minimize the risk of false discovery^4^,^5^,^24^.

In our search for cryptic MAPs, the key question was whether the all-frames database would lead to the identification of MAPs missed with the control database, which only contains the *in silico* translation of sequences assumed to be translated (for example, protein-coding transcripts). Out of 3,037 MAPs identified by the all-frames database, 2,686 MAPs were also identified by the control database among which 2,435 were unambiguously assigned to a single gene (Fig. 1b). However, the salient finding is that 351 MAPs were solely identified by the all-frames database. After these 351 putative cryptic MAPs were subjected to four stringent filtering and validation steps (see Methods), we found that 168 of them were unambiguously assigned to a single genomic region (Fig. 1b). We validated 18 cryptic MAPs using the synthetic version of them (Supplementary Fig. 2). Furthermore, we found that the Mascot score distribution (the confidence level of a peptide assignation using MS) and the transcriptomic coverage of the peptide-coding regions (PCRs) were similar for these 168 cryptic and the 2,435 conventional MAPs (Supplementary Fig. 3). It should be noted that the multiple filtering steps were designed to be particularly stringent. We therefore expect that some of the 183 discarded peptides may, nevertheless, be genuine cryptic MAPs (Fig. 1b), thereby increasing their total number up to 351 (13% of the immunopeptidome). However, at this discovery stage, we chose to conduct further analyses using only the 168 cryptic MAPs identified using our most stringent criteria (6.5% of the immunopeptidome).

### The cryptic MAPs' repertoire is linked to the HLA genotype

**Figure 2:**
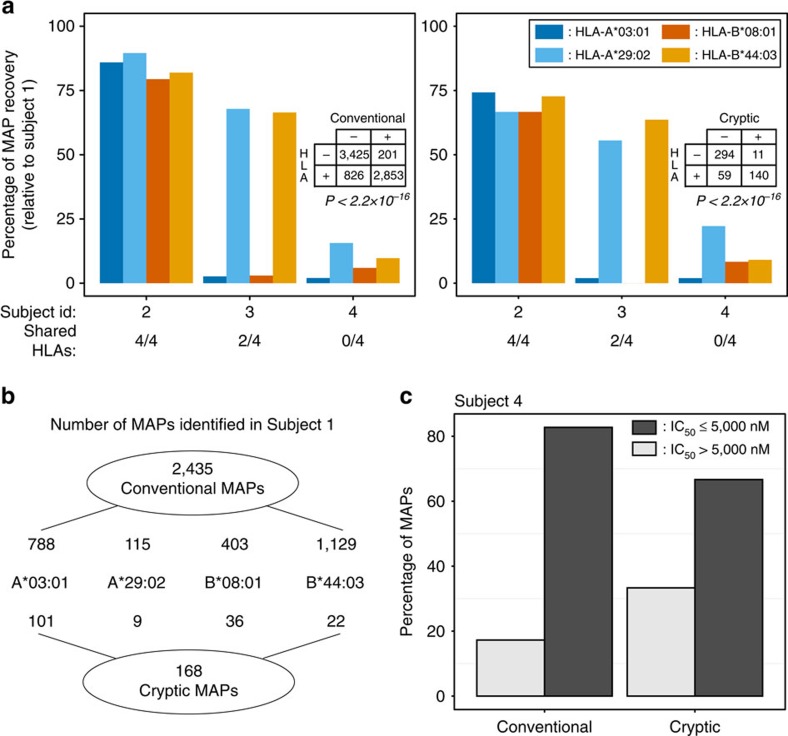
Detection of Crypt. and Conv. MAPs is HLA-dependent. (**a**) Relationship between MAP detection and HLA genotype. We sequenced MAPs on B-LCLs from three subjects who shared four, two or no HLA alleles with subject 1. We then determined the number of Conv. (left) and Crypt. (right) MAPs found in subject 1 that were shared by subjects 2–4. Each bar represents one HLA allotype. A detailed schematic of the analysis can be found in Supplementary Fig. 4. MAP detection in subjects 2–4 correlated with presence of the HLA allotype presenting the MAPs in subject 1: *P*<2.2 × 10^−16^ for Conv. and Crypt. MAPs (two-sided Fisher's exact test). (**b**) Schematic detailing of the numbers of Conv. and Crypt. MAPs identified in subject 1 for the considered HLA alleles. (**c**) Most MAPs detected in subject 4 are promiscuous binders. Overall, 168 Conv. and 9 Crypt. MAPs detected in subject 1 were also detected in subject 4, even though the two subjects did not share any HLA alleles. Using NetMHCcons, we computed the predicted binding affinity (IC_50_) of those MAPs for the four HLA-A and -B allotypes of subject 4, and we kept the lowest of the four IC_50_ values (corresponding to the highest MHC-binding affinity). The bar chart depicts the percentage of Conv. and Crypt. MAPs having an IC_50_≤ or >5,000 nM. Peptides with an IC_50_≤5,000 nM for the HLA-A/B allotypes of subject 4 were assumed to be promiscuous binders, that is, to bind subject 4 allotypes in addition to subject 1 allotypes.

Various human leukocyte antigen (HLA) allotypes have different peptide-binding motifs and therefore present different MAP repertoires. Accordingly, if a peptide eluted from cells of subject 1 is a genuine MAP, its presence on cells from other individuals should depend on the presence of the HLA allotype, presenting this peptide on cells from subject 1. In other words, the presence of authentic MAPs should be ‘HLA-restricted'. The restriction should be strong but it does not need to be perfect because there are some overlap in the MAP repertoires presented by various allotypes^25^. On the contrary, no HLA restriction should be seen between the HLA genotype and the presence of MHC-unrelated peptides. Therefore, to test whether our cryptic MAPs were HLA-restricted, we analysed the immunopeptidome of three other subjects who shared four, two or no HLA allotypes with subject 1 (Supplementary Fig. 4). For both conventional and cryptic MAPs, we found a very strong positive dependence between peptide detection in subjects 2–4 and the presence of the corresponding HLA-A or -B allotype (two-sided Fisher's exact test, *P*<2.2 × 10^−16^; Fig. 2a,b). The degree of HLA allotype restriction was similar for conventional and cryptic MAPs. Moreover, most of the MAPs detected in the absence of the relevant HLA allele were predicted to be promiscuous binders (Fig. 2c). These data further validate that cryptic peptides detected with our proteogenomic approach are genuine MAPs.

### Cryptic MAPs derive from both coding and noncoding RNAs

**Figure 3:**
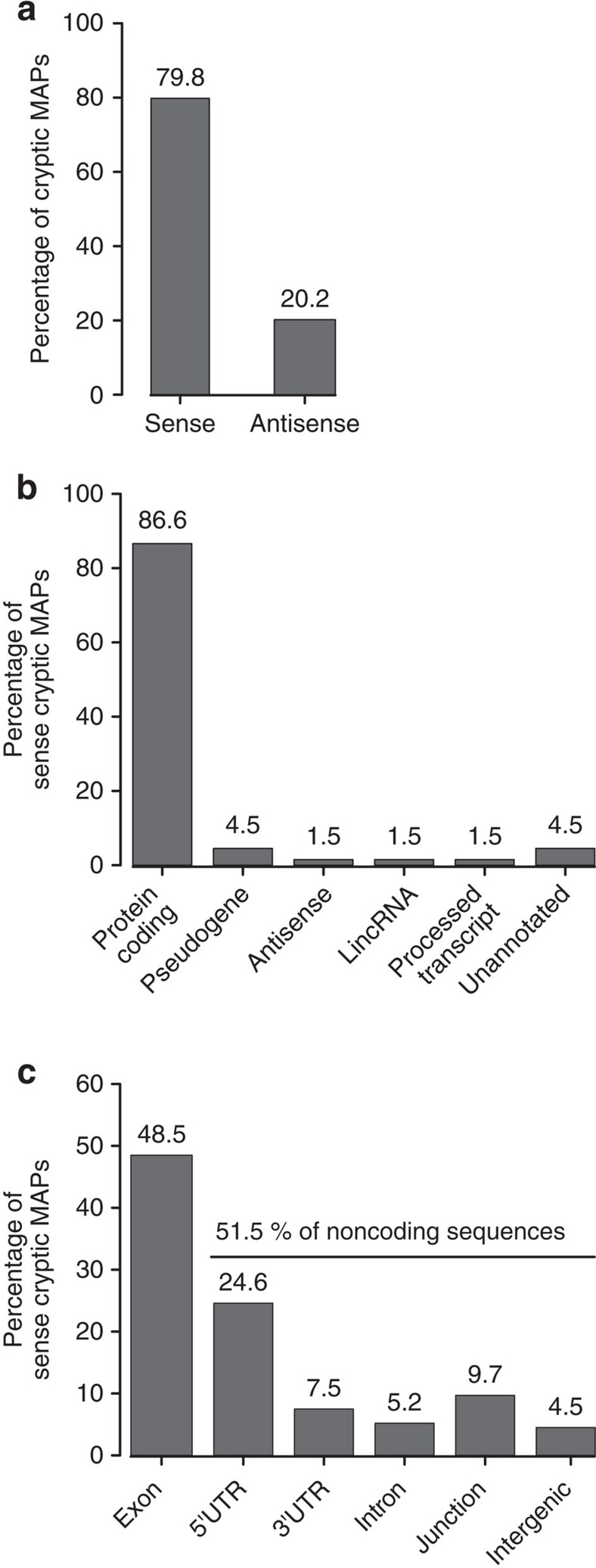
Crypt. MAPs derive from both coding and noncoding transcripts. (**a**) Some Crypt. MAPs derive from novel antisense transcripts. Bar plot showing the percentages of Crypt. MAPs derived from sense and antisense transcriptions. (**b**,**c**) For Crypt. MAPs derived from sense transcription, we determined the percentage of each gene biotype in MAP source genes (**b**) and the proportion of Crypt. MAPs generated by six types of genomic regions (**c**). The ‘exon' class refers to out-of-frame Crypt. MAPs, while the ‘junction' category corresponds to peptides encoded by intron–exon or UTR–exon junction. LincRNA, long intergenic noncoding RNAs.

Next, we analysed the origin of cryptic MAPs. A notable finding was that 20.2% of cryptic MAPs unambiguously allocated to one gene could be assigned exclusively to non-annotated antisense transcripts (transcribed from non-template DNA strand; Fig. 3a). This suggests that, although antisense transcripts are generally assumed to be noncoding^26^, their translation can generate substrates for the MHC class I antigen presentation pathway. Next, we focused our efforts on sense cryptic MAPs, as annotations were available for their respective gene source, and made two observations. First, by using the gene biotype nomenclature that classifies genes according to their biological relevance^27^, we observed that 86.6% of sense cryptic MAPs derived from protein-coding genes, 9% from genes assumed to be noncoding such as pseudogenes, annotated antisenses, long intergenic noncoding RNAs or processed transcripts and finally 4.5% from unannotated intergenic regions (Fig. 3b). Second, by analysing the location of sense cryptic MAPs within their respective gene source, we observed that 48.5% of them were produced by out-of-frame translation of exonic sequences. The remaining 51.5% originated from translation of allegedly noncoding sequences (Fig. 3c). Among those, cryptic MAPs predominantly derived from the translation of 5′UTRs as opposed to 3′UTRs (24.6% versus 7.5%). This observation is coherent with the reinitiation model for translation initiation, which implies that the probability of translation initiation decreases along the transcript^28^. A small proportion of peptides (5.2%) derived from intronic sequences, a finding consistent with a report showing that a construct coding for the model SIINFEKL peptide, could generate MAPs after insertion into an intronic sequence^29^. Finally, we observed that 9.7% of cryptic MAPs derived from UTR–exon or intron–exon junctions and thus corresponded to translation products of overlapping short ORFs or retained intron transcripts, respectively. Overall, these results highlight the complexity of the immunopeptidome by showing that the landscape of cryptic MAPs includes both sense as well as antisense coding and noncoding RNAs.

### Cryptic MAPs derive from ORFs with a 5′ end positional bias

**Figure 4:**
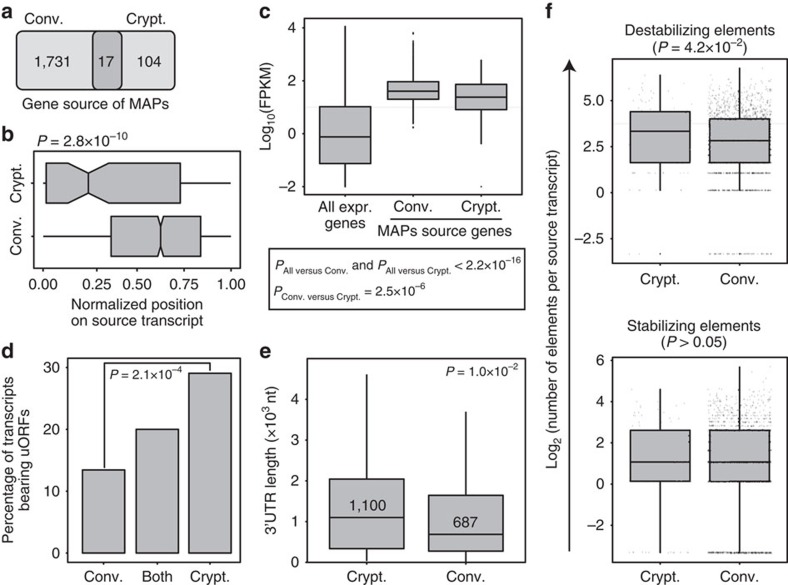
Crypt. MAPs preferentially derive from unstable mRNAs. (**a**) Venn diagram showing minimal overlap between the gene source of Conv. and Crypt. MAPs. (**b**) Crypt. MAPs preferentially derive from the 5′ end of their source transcript. The length of each source transcript was normalized to 1, and the start of each MAP was then positioned on a 0–1 scale (*x* axis), where 0 represents the 5′ end of the source transcript. Crypt. MAPs deriving from intergenic and intronic regions were excluded from this analysis. See also Supplementary Fig. 5a. (**c**) Log_10_ expression values, in FPKM, of all genes expressed in B-LCL versus the subset of the gene source of Conv. and Crypt. MAPs. (**d**) Crypt. source transcripts preferentially bear upstream ORFs (uORFs). For each MAP source transcript, we predicted the 5′UTR and 5′UTR–exon ORF initiating at an AUG embedded in an optimal or strong Kozak context. The bar graph shows the proportion of source transcripts bearing at least one uORF and generating a Conv. MAP, a Crypt. MAP or both. See also Supplementary Fig. 5b. (**e**) Crypt. source transcripts display long 3′UTRs. Using pyGeno, we retrieved the 3′UTR of MAP source transcripts (when available) and computed their length in nucleotide (nt). The boxplot displays the resulting 3′UTR length distribution for Crypt. and Conv. MAP source transcripts excluding the upper outliers that represented 6 and 107 values out of 97 and 1,770 transcripts, respectively. (**f**) 3′UTRs of Crypt. but not Conv. MAP source transcripts are enriched in destabilizing elements. We looked for destabilizing and stabilizing elements identified in ref. 36 in the 3′UTR of Crypt. and Conv. MAP source transcripts. For each source transcript, we computed the number of destabilizing and stabilizing elements contained in its sequence. The resulting distributions are plotted for Crypt. and Conv. MAP source transcripts as the log_2_ number of destabilizing (top panel) or stabilizing elements (bottom panel) per transcript. See also Supplementary Fig. 5c. Statistical significance was assessed with a two-sided (**b**,**c**,**e**) or one-sided (**f**) Wilcoxon rank sum test, or a two-sided Fisher's exact test (**d**). On box plots, boxes represent second and third quartiles, whiskers ±1.5 the interquartile range, and dots the outliers.

We next sought to determine whether specific types of genes would preferentially generate cryptic as opposed to conventional MAPs. We first noted that very few genes generated both conventional and cryptic MAPs: (i) among the 121 cryptic MAP source genes, only 17 (that is, 14%) also gave rise to conventional MAPs and (ii) only 1% of the 1,731 conventional MAP source genes generated cryptic MAPs (Fig. 4a). The small overlap between genes coding cryptic versus conventional MAP suggests that these two gene sets possess some intrinsic differential feature(s). Further analyses highlighted two conspicuous differences between genes coding conventional versus cryptic MAPs. First, cryptic PCRs were located much closer to the 5′ end of their source transcript than conventional PCRs (Fig. 4b). This shift in PCR location was observed not only for cryptic MAPs coded by 5′UTRs and 5′UTR/exons but also for the entire set of exonic cryptic MAPs (Supplementary Fig. 5a). Second, the expression level of genes coding cryptic and conventional MAPs was different. Conventional MAPs have been shown to derive preferentially from abundant transcripts^30^,^31^, and we observed that this was also the case for cryptic MAPs. However, the expression of cryptic MAP-coding genes was slightly but significantly inferior to that of conventional MAP source genes (Fig. 4c).

MAPs derive primarily from rapidly degraded proteins, and evidence suggests that the nonsense mediated decay (NMD) pathway plays a significant role in this process via translation-dependent degradation^32^,^33^. NMD targets messenger RNAs (mRNAs) containing a premature termination codon or normal mRNAs containing upstream ORFs^33^,^34^. Premature termination is predicted to result in more MAPs originating from the 5′ end of the transcript^35^, as we observed for cryptic but not conventional MAPs (Fig. 4b). In addition, we found that the proportion of MAP-coding transcripts that harboured at least one upstream ORF was significantly higher for cryptic than for conventional MAPs (30% versus 13%), while transcripts generating both types of MAPs showed an intermediate percentage (20%, Fig. 4d). Since transcripts with an upstream ORF generated cryptic MAPs from 5′UTRs but also from exons and 3′UTRs (Supplementary Fig. 5b), NMD appears to be involved in the generation of all types of cryptic MAPs. Moreover, NMD was also reported to target transcripts bearing long 3′UTRs or 3′UTRs containing intronic sequences. While the transcript source of conventional and cryptic MAPs displayed the same frequency of 3′UTR introns (Supplementary Fig. 5c), cryptic MAP source transcripts had longer 3′UTRs than conventional MAP source transcripts (1,100 versus 687 nt, Fig. 4e). Taken together, these observations suggest that NMD contributes to the generation of cryptic MAPs while lowering the abundance of cryptic MAP source transcripts relative to conventional ones (Fig. 4c) because NMD reduces the steady-state levels of its target RNAs. Besides NMD, mRNA stability is also regulated by *cis-*regulatory elements that are located in 3′UTRs and interact with RNA-binding proteins^36^. In line with this, relative to conventional MAP source transcripts, the 3′UTRs of cryptic MAP source transcripts contained similar numbers of stabilizing elements but an increased number of destabilizing elements (Fig. 4f). In other words, cryptic MAP source transcripts display longer 3′UTRs with a selective enrichment in destabilizing elements. Taken together, our data suggest that cryptic MAPs derive from unstable transcripts targeted by NMD or 3′UTR-destabilizing elements.

### Cryptic MAPs derive from precursors with atypical C termini

**Figure 5:**
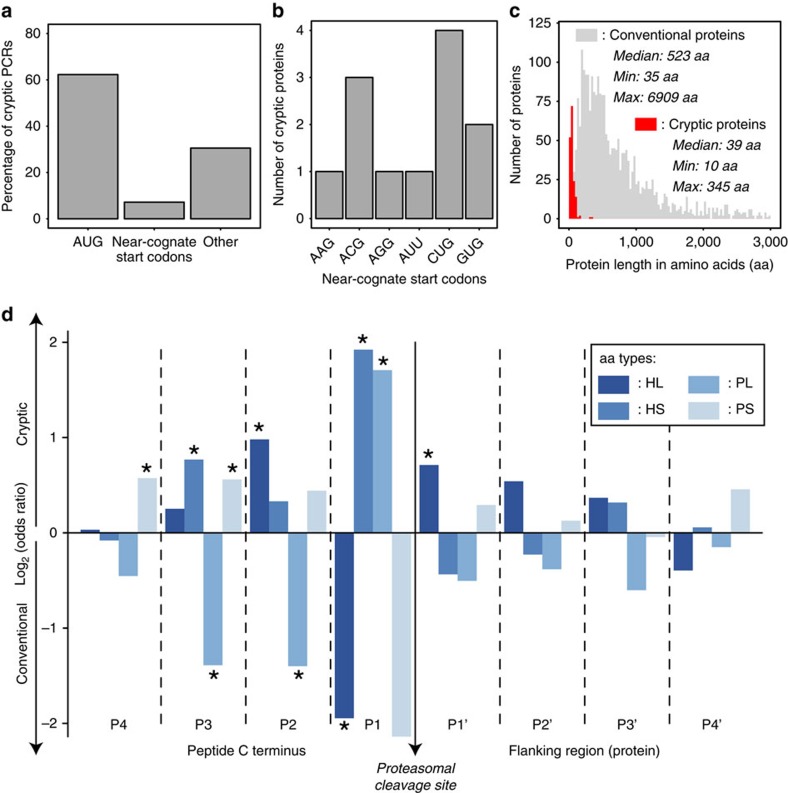
Features of ORFs coding Crypt. MAPs. (**a**) Most Crypt. PCRs are in-frame with an upstream start codon. To predict the probable start codon of each Crypt. PCR, we sequentially applied the following rules: (i) presence of an upstream AUG within an optimal (GCC[R]CCstartG[V]), strong ([R]NNstartG[V]) or weak (anything else) Kozak context, (ii) presence of an upstream near-cognate start codon within an optimal or strong Kozak context, (iii) any other codon downstream of the first upstream stop codon. Bars represent the percentage of Crypt. PCRs displaying an upstream in-frame AUG, near-cognate start codon or any other codon as a probable initiation codon. (**b**) Bar plot showing near-cognate start codon usage at putative translational start sites of 12 Crypt. source proteins. (**c**) Length distribution of Conv. and predicted Crypt. proteins. Median, minimum (Min) and maximum (Max) observed lengths are indicated on the graph for both types of proteins. Conv. proteins having a length >3,000 amino acids are not displayed on the graph. (**d**) Crypt. and Conv. MAPs do not have the same amino-acid composition at their C termini. Amino acids (aa) were classified in four categories: Hydrophobic/Large (HL), Hydrophobic/Small-Medium (HS), Polar/Large (PL) and Polar/Small-Medium (PS)^44^. For the MAP C terminus (positions P4 to P1) and its C-terminal flanking region (positions P1′ to P4′), we compared the usage of those four aa categories at each position between Crypt. and Conv. MAPs. The graph displays the log_2_(odds ratio) and significant differences are marked with an asterisk (*P<0.05; two-sided Fisher's exact test).

To gain further insights into the mechanisms responsible for the generation of cryptic MAPs, we analysed the nucleotide sequence of MAP source transcripts to predict their translation start and stop sites. Notably, we observed that translation initiation occurred at a known initiation codon for 69% of cryptic MAPs: AUG was used more often than near-cognate start codons, which differ from AUG by a single nucleotide (62% versus 7%). This suggests that, even for those atypical proteins, AUG is the preferential translation initiation codon (Fig. 5a). Among near-cognate start codons, CUG was the most commonly observed (Fig. 5b). This observation is in agreement with several reports demonstrating that CUG is the most efficient near-cognate start codon to initiate translation^18^,^37^,^38^. Other near-cognate start codons that were used more than one time included ACG and GUG, which were both shown to be enriched at translation initiation sites by ribosome profiling^38^. Finally, 31% of cryptic MAPs did not display any of the known translation initiation codons upstream of their respective PCR (Fig. 5a). In accordance with similar observations based on analyses of ribosome-profiling data^38^, these data suggest that translation can be initiated at other codons than the classical AUG or near-cognate start codons.

The median length of conventional proteins is ∼400 amino acids and, simply by virtue of their size, longer proteins generate more MAPs than shorter proteins^14^. Accordingly, the median length of conventional MAP source proteins in our data set was 523 amino acids. In stark contrast, the median length of cryptic MAP source proteins was 39 amino acids, and 75% of them had less than 62 amino acids (Fig. 5c). The shortest predicted cryptic proteins (3 out of 168) had a length of 10 amino acids and generated cryptic MAPs of 9 amino acids; MHC processing of these cryptic MAPs only required trimming of the N-terminal methionine. The generation of conventional MAPs is initiated by proteasomal cleavage followed in general by exopeptidase trimming of the N terminus but not the C terminus^39^,^40^,^41^. Therefore, with few exceptions, the C terminus created by the proteasome remains intact in conventional MAPs^42^,^43^. Given the remarkably short size of cryptic MAP source proteins, we hypothesized that many cryptic MAPs may not need proteasomal degradation before entering the MHC class I antigen presentation pathway. We reasoned that, if cryptic MAPs were proteasome-independent, their C terminus might be different from that of (proteasome-dependent) conventional MAPs. To test this hypothesis, we analysed amino-acid usage at the four C-terminal amino acids of individual MAPs and the four amino acids downstream of the C terminus (in the source protein) for conventional versus cryptic MAPs. The 20 amino-acid residues were grouped into four categories based on their bulkiness and hydrophobicity^44^, and we analysed these data to determine which categories were enriched or depleted at each position for the two types of MAPs. We found that, out of the eight considered positions, five displayed significant differential amino-acid class usage between cryptic and conventional MAPs (Fig. 5d). Together, the facts that cryptic MAPs originate from very short proteins and that amino-acid usage around their C termini is different from that of conventional MAPs suggest that processing of cryptic MAPs may be proteasome-independent.

### Cryptic MAPs display distinct features and are immunogenic

**Figure 6:**
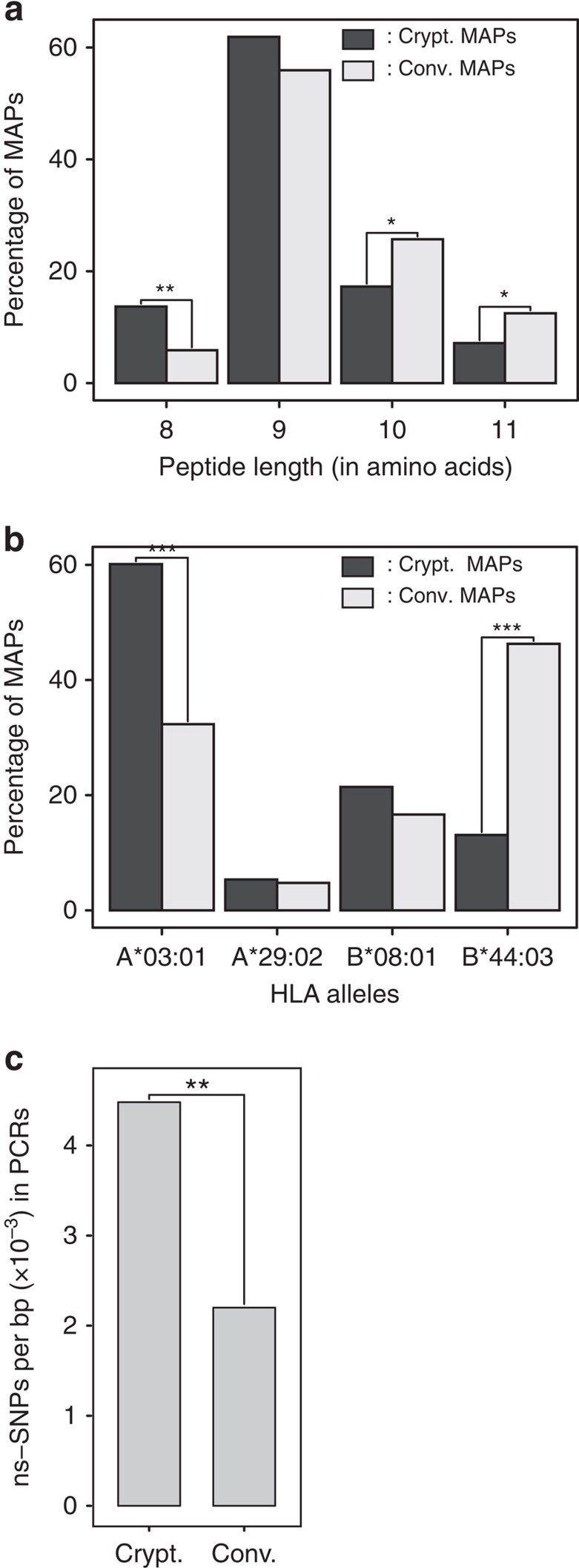
Crypt. and Conv. MAPs display different features. (**a**–**c**) Bar plots showing that Crypt. and Conv. MAPs from subject 1 have different (**a**) length distribution, (**b**) allotype distribution and that (**c**) their PCRs exhibit different ns-SNP frequencies (from dbSNP138). In all cases, statistical significance was assessed using a two-sided Fisher's exact test: *P≤0.05, **P≤0.006, ***P≤1.10^−11^ in the bar plots.

We next evaluated relevant structural and functional features of cryptic MAPs *per se*. Relative to conventional MAPs, we found that cryptic MAPs exhibited three distinctive characteristics: they were shorter, had different allotype-binding preferences and harboured more genomic polymorphisms (Fig. 6). The length distribution of cryptic MAPs revealed a significant enrichment in 8-mers and depletion in 10–11-mers when compared with conventional MAPs (Fig. 6a). This further supports the idea that cryptic and conventional MAPs are processed differently by peptidases. Unexpectedly, we found that cryptic MAPs were preferentially presented by HLA-A*03:01, while conventional MAPs were preferentially presented by HLA-B*44:03 in subject 1 (Fig. 6b). Proteogenomic studies of MAPs presented by other HLA allotypes will be required to assess whether differential allotype preferences of cryptic and conventional MAPs can be generalized. If it were the case, one implication would be that the HLA genotype dictates the breadth of the cryptic immunopeptidome presented at the cell surface. No bias in favour or against ns-SNPs was found in conventional MAP PCRs^22^. However, we found that cryptic MAP PCRs contained a significantly higher frequency of ns-SNPs than conventional MAP PCRs (Fig. 6c; *P*=5.625 × 10^−3^). In other words, cryptic MAPs derive from genomic sequences that are more polymorphic at the population level than conventional protein-coding sequences.

**Figure 7:**
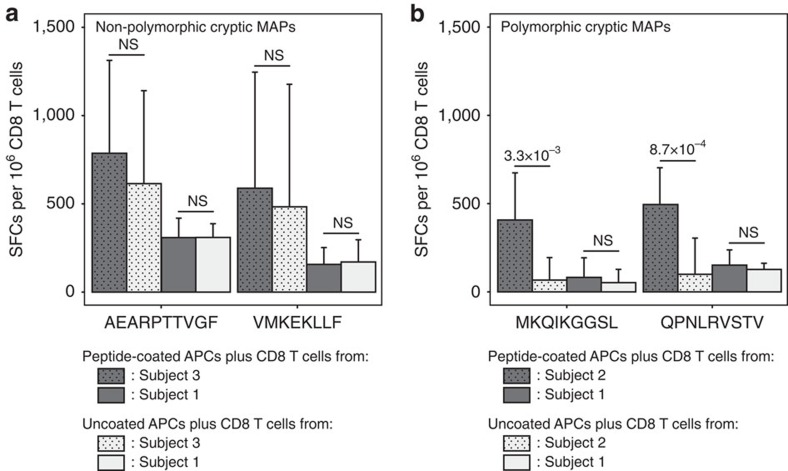
Immunogenicity of Crypt. MAPs. (**a**,**b**) Only polymorphic Crypt. MAPs are immunogenic. IFN-γ Elispot counts showing the number of spot-forming cells (SFCs) per million CD8 T cells for two non-polymorphic (**a**) and two polymorphic (**b**) Crypt. MAPs. Final counts were obtained following the subtraction of background spots (peptide-coated APCs alone) from the spots obtained when CD8 T cells were exposed to peptide-coated or uncoated APCs. The experiment was performed in biological triplicates (each with three technical replicates), error bars represent s.d. and statistical significance was assessed using a two-tailed Student's *t*-test (NS: not significant, *P*>0.05). Features of the four tested Crypt. MAPs are detailed in Tables 1 and 2.

Finally, we wished to determine whether cryptic MAPs could be immunogenic. To this end, we studied the T-cell response of subjects 2 and 3 against four randomly selected cryptic MAPs, whose sequence was validated using synthetic peptides (Supplementary Fig. 2a–d), and that were not detected on their own B-LCLs but were present on B-LCLs from subject 1. Two of these MAPs were present on B-LCLs from subject 1 but not subject 2 (HLA-identical to subject 1) because of an unshared ns-SNP in the genomic sequence coding for these MAPs (Table 1). Two other MAPs were detected in subject 1 but not subject 3, presumably because of an unidentified *trans*-acting factor since the MAP-coding transcripts and the relevant HLA allotypes were expressed in both subjects (Table 2). Peripheral blood mononuclear cells (PBMCs) from subject 1, 2 or 3 were co-cultured with autologous monocyte-derived dendritic cells (DCs) pulsed with one of the four cryptic MAPs (synthetic peptides). After culture for 12 days in the presence of interleukin (IL)-7 and IL-15, cells were harvested and CD8^+^ cells were separated from CD8^−^ cells using FACS. Elispot was then used to quantify interferon (IFN)-γ-producing cells in wells containing either CD8 T cells alone or together with peptide-pulsed or -unpulsed CD8^−^ APCs. Non-polymorphic MAPs did not elicit a MAP-specific response (Fig. 7a). However, polymorphic MAPs elicited a MAP-specific response since the frequency of IFN-γ-producing cells was much higher in the presence of peptide-pulsed than -unpulsed APCs (Fig. 7b). We conclude that, at least *in vitro*, polymorphic cryptic MAPs can be immunogenic.

## Discussion

The present work demonstrates that proteogenomics can provide a systems-level perspective on the landscape of the cryptic immunopeptidome. The fact that a sizeable proportion of MAPs are cryptic (6.5–13% depending on stringency criteria) enhances the complexity of the immunopeptidome. If anything, we might have underestimated the proportion of cryptic MAPs in the immunopeptidome because our RNA-seq was performed on poly(A) tailed RNAs. The prevailing dogma holds that polyadenylation of RNA precursors is required for nuclear export and stability of mature transcripts and for efficient translation of mRNAs^45^. However, recent reports suggest that immature mRNA precursors can be translated in the nucleus and generate MAPs^29^,^46^. Further proteogenomic studies will therefore be needed to assess the potential contribution to the MAP repertoire of RNAs without poly(A) tail. In addition, RNA-seq-based proteogenomic studies may miss the rare MAPs derived from non-contiguous protein sequences via proteasome-mediated splicing^14^.

About 50% of cryptic MAPs result from out-of-frame translation and the other half from translation of allegedly noncoding sequences. The ultimate biological role of cryptic translation remains elusive. However, it might be unwise to assume that this phenomenon merely represents ‘translational noise'. Protein synthesis is demanding: it is the most energy-consuming process in the cell as it monopolizes 45% of cellular ATP supplies^47^. Furthermore, any RNA sequence subject to translation will experience selection against encoding a protein with detrimental impact on cell function^6^. In any case, noncoding RNAs are vital, and our demonstration that several noncoding RNAs generate MAPs means that CD8 T cells have an opportunity to scrutinize these transcripts.

The gene source of conventional MAPs are enriched in microRNA-binding elements, suggesting that mRNA destabilization favours MAP generation^30^. By comparing transcripts coding conventional and cryptic MAPs, we obtained meaningful evidence suggesting that cryptic MAPs derive from particularly unstable transcripts targeted by NMD or 3′UTR destabilizing elements: (i) cryptic MAP transcripts were enriched in upstream ORFs and their PCRs showed a strong 5′ end positional bias (suggestive of premature termination) and (ii) cryptic MAP transcripts displayed longer 3′UTR enriched in destabilizing but not stabilizing elements when compared with conventional source transcripts. Together with previous work by us and others, these data allow for the development of an emerging model in which mRNA instability is instrumental in the genesis of all types of MAPs. This model is an extension of the idea that most MAPs derive from defective ribosomal products^32^,^48^,^49^: unstable RNAs targeted by NMD, microRNAs or other 3′UTR-destabilizing elements would generate more defective ribosomal products and therefore more MAPs. The validity of this model can be submitted to high-throughput experimental validation: if it is correct, mRNA half-life should be negatively correlated to MAP generation. We do not exclude that translation efficiency, which partly depends on codon usage^50^, might also regulate MAP generation. Indeed, although we did not find evidence for a codon bias in conventional source transcripts versus cryptic MAP source ORFs (*P*=0.34, odds ratio=1.02), we observed that MAP source transcripts or ORFs in general use rare codons slightly more frequently than transcripts that do not generate MAPs (*P*<2.2 × 10^−16^, odds ratio=1.14; Supplementary Tables 2 and 3). Therefore, it might be interesting to further investigate the impact of codon bias on MAP generation.

Some 25 years ago, Boon and van Pel^51^ proposed that MAPs might derive in a proteasome-independent manner from translation of short subgenic regions (peptons). This unorthodox hypothesis has progressively fallen into disfavour because no such MAPs were discovered with MS^39^. The present work argues that such MAPs do exist but can, in practice, be detected only by proteogenomics. Indeed, our cryptic MAPs were coded by extremely short ORFs, and the amino-acid composition of their C termini suggests that they are, at least in part, proteasome-independent.

One area where cryptic MAPs may be most relevant is cancer immunology. Although the vast majority of cancer mutations involve non-exomic regions, searches for tumour-specific antigens (TSAs) have focused on exomic mutations^31^,^52^,^53^,^54^. Nonetheless, since numerous noncoding transcripts are expressed only in cancer cells^55^,^56^, a number of cryptic MAPs may be genuine TSAs. Furthermore, we demonstrated that (i) cryptic MAP PCRs displayed a higher frequency of germline polymorphisms (ns-SNPs) than the conventional exome (Fig. 6c) and that (ii) polymorphic cryptic MAPs discovered by proteogenomics were immunogenic (Fig. 7b). Hence, it is reasonable to expect that cryptic MAPs bearing somatic mutations (that is, TSAs) should also be immunogenic. Accordingly, in melanoma and renal cell carcinoma, pioneering studies using more traditional approaches have uncovered unique immunogenic cryptic TSAs derived from noncoding regions^19^,^21^. Assuming that cryptic MAPs may be a rich source of heretofore overlooked TSAs, it is imperative to directly explore the presence of cryptic TSAs using systems-level approaches. Expanding the repertoire of TSAs would be highly beneficial because the low number of immunogenic exome-derived TSAs is a major hurdle for cancer immunotherapy^57^,^58^,^59^.

## Methods

### Subject recruitment

Written informed consent was obtained from all study participants. The study protocol was approved by the Comité d'Éthique de la Recherche de l'Hôpital Maisonneuve-Rosemont. Relative to subject 1 (HLA-A*03:01, -A*29:02; -B*08:01, -B*44:03), subjects 2–4 were HLA-identical, HLA-haploidentical (HLA-A*02:01, -A*29:02; -B*57:01, -B*44:03) or HLA-disparate (HLA-A*01:01, -A*02:01; -B*18:01, -B*39:24). See also Supplementary Table 1.

### Analysis of RNA-seq data

RNA-seq was performed as described^22^. Paired-end RNA-seq data of subject 1 were mapped on the human reference genome (GRCh37.75) with the Casava 1.8.1 and Eland v2e mapping softwares (Illumina). This alignment was used to perform SNP calling with the Casava 1.8.1 software as previously described^22^. Only ns-SNPs having a Qmax_gt≥20 were used to build the customized control database.

To obtain an expression value for each transcript of a given gene, paired-end RNA-seq data from subject 1 were mapped on the reference genome (GRCh37.75) using TopHat 2.0.10 (ref. 60). Cufflinks 2.2.1 (ref. 61) was then run on the output-sorted BAM file in addition to the Ensembl gtf file to obtain FPKM (fragment per kilobase of transcript per million mapped reads) values for all known transcripts. Only transcripts having an FPKM value >0 were considered as expressed.

### Generation of the control and all-frames databases

We generated two customized databases based on the RNA-seq data of subject 1. To generate the control database, we applied a workflow similar to the one of Granados *et al.*^22^: ns-SNPs identified in subject 1 were integrated at their correct position in the reference genome (GRCh37.75) to build a personalized genome. Using the Ensembl gtf file, we extracted all known transcripts and further *in silico* translated them in their canonical reading frame to obtain the canonical proteome of subject 1. To generate the all-frames database, we used all reads passing the Illumina quality filters and *in silico* translated them in the six possible reading frames using a sliding window of 33 bp to obtain all theoretical peptides having a length between 8 and 11 amino acids. For each peptide, we computed an *S*-value, that is, the number of times it was seen following the *in silico* translation process. Only peptides having an *S-*value ≥10 as well as a length between 8 and 11 amino acids were included in the predicted peptidome of subject 1. Both the canonical proteome and the predicted peptidome of subject 1 were compiled in fasta files to obtain the control and the all-frames database, respectively. Both databases were then concatenated with their respective decoy counterpart and submitted to the Mascot database search engine along with subject 1's immunopeptidomic data.

### MS analyses

Immunopeptidomics raw data from subjects 1 and 2 B-LCL were obtained from a previous study^30^. For subjects 3 and 4, MAPs were eluted from B-LCLs and sequenced using MS as previously described (three to four biological replicates per subject)^22^. Each replicate was separated in six fractions using strong cation exchange chromatography. Vacuum-dried fractions were then suspended in 5% acetonitrile and 0.2% formic acid and injected into the LTQ-Orbitrap Elite operating at a resolving power of 60,000 (at *m*/*z* 400) for both full spectra and MS/MS spectra modes. Up to 10 precursor ions were accumulated to the target value of 50,000 with a maximum injection time of 100 ms. Mass spectra were analysed using the Xcalibur software and peak lists were generated with Mascot Distiller.

### Control and all-frames database searches

The Mascot search engine (Matrix Science) was used in combination with the control or the all-frames database concatenated to their reverse database to identify peptides present in the immunopeptidome of subject 1. Mass tolerances on precursor and fragment ions were set to 5 p.p.m and 0.02 Da, respectively. Searches were performed without enzyme specificity, and cysteinylation, phosphorylation (on Ser, Thr and Tyr), oxidation (Met) and deamidation (Asn, Gln) were used as variable modifications. Following each database search, we converted raw files to peptide maps containing *m*/*z* values, charge state, retention time and intensity above detection threshold (≥8,000) using ProteoProfile (http://proteomics.iric.ca/tools/ProteoProfile/)^62^. The peptide maps were used to extract the abundance of the identified peptides across the four replicates.

On the 8–11 amino-acid-long peptides identified with the control database, we computed the FDR^63^ for all combinations of the Mascot score (which represents the confidence level of a peptide assignation) and predicted MHC-binding affinity (computed with NetMHCcons^64^). FDRs were computed as (number of decoy identifications/number of target identifications) × 100. We then selected the combination of the Mascot score and MHC-binding affinity yielding the higher number of MAPs at 5% FDR, as described^22^. The same Mascot score (≥22) and MHC-binding thresholds (≤1,250 nM) were then applied to the peptide list identified with the all-frames database. As expected, considering the unavoidable effect of database size on FDRs calculated according to decoy approaches^5^,^24^,^65^, applying the thresholds defined with the control database to the all-frames database increased the decoy-based FDR to 9% for the all-frames database.

### Identification of cryptic and conventional MAPs

Peptides identified with both the control and the all-frames databases were considered as conventional MAPs. Peptides solely identified by the all-frames database were considered as putative cryptic MAPs. To validate whether they were genuine cryptic MAPs, we mapped the subset of peptide-encoding reads using TopHat to discard peptides coming from multiple locations in the genome. The remaining cryptic MAP candidates were assigned to their respective source gene and their MS/MS spectra were manually validated. To determine the type of sequence (within the source gene) generating each cryptic MAP, we used the intersect function of the BEDTools suite on the bed file of our cryptic candidates as well as Ensembl gtf file. Peptides assigned to a gene source in the opposite orientation were classified as antisense cryptic MAPs, those deriving from noncoding RNAs, 5′UTR, intronic, 3′UTR or intergenic sequences were classified as sense noncoding cryptic MAPs. Peptides deriving from exons of protein-coding genes were subjected to a reading frame validation: only peptides produced by non-canonical reading frames were classified as sense coding cryptic MAPs. For sense cryptic MAPs (except intergenic ones), we retrieved the gene biotype of their respective gene source from Ensembl annotations (when available) using pyGeno. Finally, since MAPs derive preferentially from highly abundant transcript^30^,^31^,^66^, we assumed that the conventional and sense cryptic MAPs passing all of our filtering steps were generated by the most highly expressed isoform of their respective source gene. A complete list of identified conventional and cryptic MAPs can be found in Supplementary Data 1 and Supplementary Data 2, respectively.

### Computation of PCR coverage

We computed the coverage of all identified PCRs by using the coverage function of the BEDTools suite. The sorted BAM file obtained following the TopHat alignment as well as the bed files of our cryptic and conventional PCRs were used as entry files. This coverage metrics, which represents the number of reads overlapping, by at least 1 bp, our PCRs were then correlated with the *S*-value metrics, which approximates the number of read fully overlapping the same PCRs (Supplementary Fig. 1a).

### Influence of the HLA genotype on the MAP repertoire

The Mascot search engine was used to perform database searches on the raw data of subjects 2–4 against a validation database that contained all identifications made in subject 1 as well as their decoy sequences. Mass tolerances on the precursor and fragment ions were set to 5 p.p.m and 0.02 Da, respectively. Peptide lists identified in each subject were extracted and compared with the 2,435 conventional and 168 cryptic MAPs identified in subject 1 (Supplementary Fig. 4).

### Prediction of upstream ORFs

For each transcript source of MAPs, we extracted the personalized mRNA sequences of subject 1 using pyGeno. We scanned the transcript from its 5′end to its 3′end to predict all possible ORFs initiating at an AUG embedded in an optimal (GCC[R]CCstartG[V]) or strong ([R]NNstartG[V]) Kozak context. ORFs located in the 5′UTR or at the 5′UTR–exon junction were considered as upstream ORFs. We computed the proportion of the transcript source of cryptic and/or conventional MAPs that presented at least one upstream ORF. Statistical significance between the cryptic and conventional source transcript categories was assessed using a two-sided Fisher's exact test. This analysis was performed on sense cryptic MAPs for which a source gene and transcript were available.

### mRNA stability analysis

Using pyGeno, we retrieved the 3′UTR sequences of cryptic and conventional source transcripts to compute their length, their number of intronic sequences and to look for exact match of all destabilizing and stabilizing elements characterized by Zhao W. *et al.*^36^ The 3′UTR length distributions as well as the number of destabilizing and stabilizing elements per transcript were compared between the transcript source of conventional and cryptic MAPs. Statistical significance was assessed using a two- and a one-sided Wilcoxon rank sum test, respectively. Statistical significance for the proportion of conventional and cryptic MAP source transcripts containing no versus at least one intron was assessed using a two-sided Fisher's exact test. This analysis was performed on sense cryptic MAPs for which a source gene and transcript were available.

### Prediction of cryptic source proteins

To predict the probable start codon of each cryptic PCR, we sequentially applied the following rules: (i) presence of an upstream AUG within an optimal (GCC[R]CCstartG[V]), strong ([R]NNstartG[V]) or weak (anything else) Kozak context, (ii) presence of an upstream near-cognate AUG within an optimal or strong Kozak context, (iii) any other codon downstream of the first upstream stop codon. The probable stop codon was assumed to be the first in-frame stop codon downstream of the PCR. This analysis was performed on personalized mRNA sequences of cryptic source transcripts for most sense cryptic MAPs. Since no gene structures were known for antisense, intronic and intergenic cryptic MAPs, we simply extracted the personalized genomic sequences flanking the PCR (750 bp long) and performed the same analysis.

### C-terminal amino-acid signature

At each position analysed, we compared the usage of each amino-acid class between cryptic and conventional MAPs using a two-sided Fisher's exact test. Hits were considered significant when they yielded a *P* value<0.05.

### ns-SNP frequency analysis

We used dbSNP138 (common_all set) to determine the frequency of ns-SNPs, at the population level, in the PCRs of conventional and cryptic MAPs. Since some cryptic MAPs derive from out-of-frame exonic translation, we could not rely on the synonymous versus non-synonymous dbSNP annotations. To circumvent this problem, we sequentially inserted all SNPs intersecting with our cryptic and conventional PCRs (stored in bed files). Those mutated PCRs were then *in silico* translated. If the resulting peptide was identical to the MAP initially identified in subject 1, the SNP was classified as synonymous. Otherwise, the SNP was classified as non-synonymous. Knowing the number of bp encoding our cryptic and conventional MAPs, we computed the frequency of ns-SNPs per bp observed in both types of PCRs. Statistical significance was assessed using a two-sided Fisher's exact test.

### Rare codon usage analysis

Codons were classified as rare and common if their observed usage frequency (http://www.genscript.com/cgi-bin/tools/codon_freq_table)^67^ was lower and greater than their expected usage frequency (1/number of codons encoding a given amino acid), respectively. Out of 64 codons, 30 were classified as rare and 34 as common. Using an in-house python script, we computed the number of occurrence for each codon to further derive the number of rare and common codons used by each class of transcripts across (1) conventional source transcripts versus cryptic source ORFs and (2) MAP source transcripts or ORFs versus all the other transcripts for which a cDNA sequence was defined. Statistical significance was assessed using a two-sided Fisher's exact test.

### T-cell priming and IFN-γ Elispot assays

Monocyte-derived DCs were generated from frozen PBMCs, as previously described^68^. Peptide-specific CD8+ T cells were expanded as described, with some minor modifications^69^. Briefly, thawed PBMCs were first T-cell-enriched using the Easysep Human T Cell Enrichment Kit (StemCell Technologies) and co-incubated with autologous peptide-pulsed DCs at a DC:T cell ratio of 1:4 with the addition of IL-21 (30 ng ml^−1^). Cells were cultured in CellGro DC medium containing 5% human serum and L-glutamine. IL-15 (2.5 ng ml^−1^) and IL-7 (2.5 ng ml^−1^) were added on day 3 and every 3 days thereafter. On day 12, cells were harvested and stained with an anti-human CD8-PE as recommended by the manufacturer (clone RPA-T8, BD Biosciences). CD8^+^ T and CD8^–^ cells were sorted using a FACSAria apparatus and then used for the Elispot assays, which were performed as described^70^. IFN-γ production was expressed as the number of peptide-specific spot-forming cells per 10^6^ CD8^+^ T cells after subtracting the spot counts from negative control wells (CD8 T cells alone).

### Data analysis and visualization

Unless stated otherwise, analyses were performed using the pyGeno python package (https://github.com/tariqdaouda/pyGeno)^23^. The ggplot2 package from the R software was used for data visualization. All codes are available on request to the corresponding author.

## Additional information

**Accession codes:** RNA-seq data for the four subjects are available in the Gene Expression Omnibus database under accession code GSE67174 (http://www.ncbi.nlm.nih.gov/geo/query/acc.cgi?token=upkvweysnxabzkr&acc=GSE67174). MS data are available in PeptideAtlas for subjects 1 and 2 (http://www.peptideatlas.org/PASS/PASS00270); data from subjects 3 and 4 have been submitted to the ProteomeXchange Consortium^68^ via the PRIDE partner repository with the data set identifiers PXD001898 (project accession) and 10.6019/PXD001898 (project DOI). In addition, the entire list of MAPs identified in subject 1 has been deposited into the Immune Epitope Database (http://www.iedb.org) under accession code 1028836.

**How to cite this article:** Laumont, C.M. *et al.* Global proteogenomic analysis of human MHC class I-associated peptides derived from non-canonical reading frames. *Nat. Commun.* 7:10238 doi: 10.1038/ncomms10238 (2016).

## Supplementary Material

Supplementary Information Supplementary Figures 1-5 and Supplementary Tables 1-3

Supplementary Data 1 List of all conventional MAPs detected in subject 1. Table presenting the genomic and proteomic features of all conventional MAPs.

Supplementary Data 2 List of all cryptic MAPs detected in subject 1. Table presenting the genomic and proteomic features of all cryptic MAPs. The Cryptic_status column shows from which region a cryptic MAP derives: 5 UTR, 5 UTR/EXON, EXON, INTRON/EXON, INTRON, RETAINED INTRON and 3 UTR for MAPs assigned to a known transcript; ANTISENSE for MAPs deriving from a potential novel antisense transcript overlapping a known transcript and INTERGENIC for MAPs that were not assigned to any known transcripts. The Peptide_reading_frame column indicates if partially or fully exonic cryptic MAPs are in-frame or not (In/Out) with the known reading-frame of their respective source transcript. NO FRAME or NA otherwise. Finally, the Nterminal_extension column determines if 5 UTR and 5 UTR/EXON MAPs derive (or not) from a potential N-terminal extension of their respective source transcript (TRUE/FALSE, NA for any other cryptic status).

## Figures and Tables

**Table 1:** Features of polymorphic cryptic MAPs presented in Fig. 7.

**Polymorphic MAPs**	**Cryptic status**	**HLA**	**IC**_**50**_ **(nM)**	**Subject 1**	**Subject 2**
I/MKQIKGGSL	Novel antisense	B*08:01	(I) 5,071.92/(M) 335.50	I/M	I
QPNF/LRVSTV	Exon—out	B*08:01	(F) 739.13/(L) 784.45	F/L	F

HLA, human leukocyte antigen; IC_50_, half-maximal inhibitory concentration; MAP, MHC class I-associated peptide; MHC, major histocompatibility complex; MS, mass spectrometry.

The columns Subject 1 and Subject 2 indicate the peptide variant coded by transcripts found in each subject as well as a positive MS detection when the amino acid is underlined.

**Table 2:** Features of non-polymorphic cryptic MAPs presented in Fig. 7.

**Non-polymorphic MAPs**	**Cryptic status**	**HLA**	**IC**_**50**_ **(nM)**	**Subject 1**	**Subject 3**
AEARPTTVGF	Exon—out	B*44:03	119.38	AEA	AEA
VMKEKLLF	Intron	A*29:02	883.60	VMK	VMK

HLA, human leukocyte antigen; IC_50_, half-maximal inhibitory concentration; MAP, MHC class I-associated peptide; MHC, major histocompatibility complex; MS, mass spectrometry.

The columns Subject 1 and Subject 3 indicate the peptide variant coded by transcripts found in each subject as well as a positive MS detection when the amino acid is underlined.
