# Supplementary material for: Global proteogenomic analysis of human MHC class I-associated peptides derived from non-canonical reading frames
Source: Nat Commun. 2016 Jan 5;7:10238. doi: 10.1038/ncomms10238 (PMC4728431; doi:10.1038/ncomms10238)
Supplement: Supplementary Information — Supplementary Figures 1-5 and Supplementary Tables 1-3 [file ncomms10238-s1.pdf]

SUPPLEMENTARY FIGURES

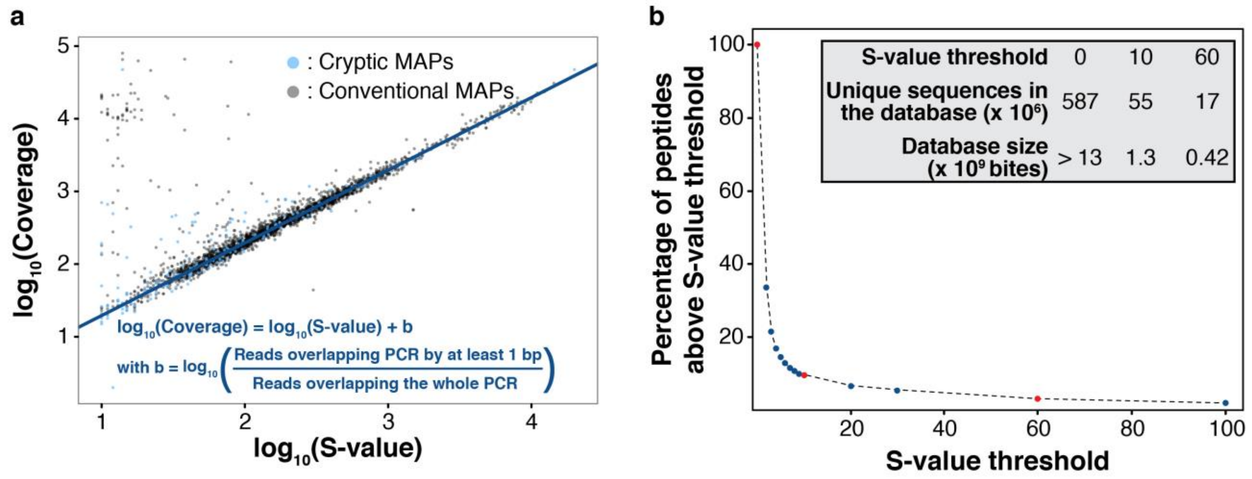

Supplementary Figure 1. Selection of the S-value threshold. (**a**) The S-value metrics correlates with the coverage. Following RNA-seq reads mapping on the reference genome (version GRCh37.75) using TopHat, we computed the coverage of each peptide-coding region (PCR). For each identified MAP, we then plotted this coverage metrics as a function of the S-value, both in $\log_{10}$. Since the S-value metrics approximates the number of reads spanning the whole PCR while the coverage also takes into account reads spanning PCR by at least one base pair (bp), we reasoned that the coverage would slightly overestimate the S-value. For a few MAPs, the S-value appeared to underestimate the coverage: analysis of those MAPs revealed that most of them derived from genes in the RPS and RPL families. Highly similar in sequence but not identical, these genes will generate many 11-amino acids entries in the all-frames database, having different S-value but all containing the 9-amino acids MAPs of interest. Since we kept only one S-value among all possible ones, the real S-value of this 9-mers was therefore a strong underestimation of the coverage given by TopHat especially since this mapper maps multihit reads. (**b**) An S-value threshold ≥ 10 yields a database having a size manageable by Mascot search engine. This graph represents the percentage of peptides above the S-value threshold as a function of the S-value threshold. Number of unique sequences in the database as well as its size (in bites) were computed for three S-value thresholds (red dots) as detailed in the table. For comparison, the size of a typical reference protein database, such as UniProt, is about 0.5×10^9 bites.

a

Endogenous peptide (related to Fig. 7)

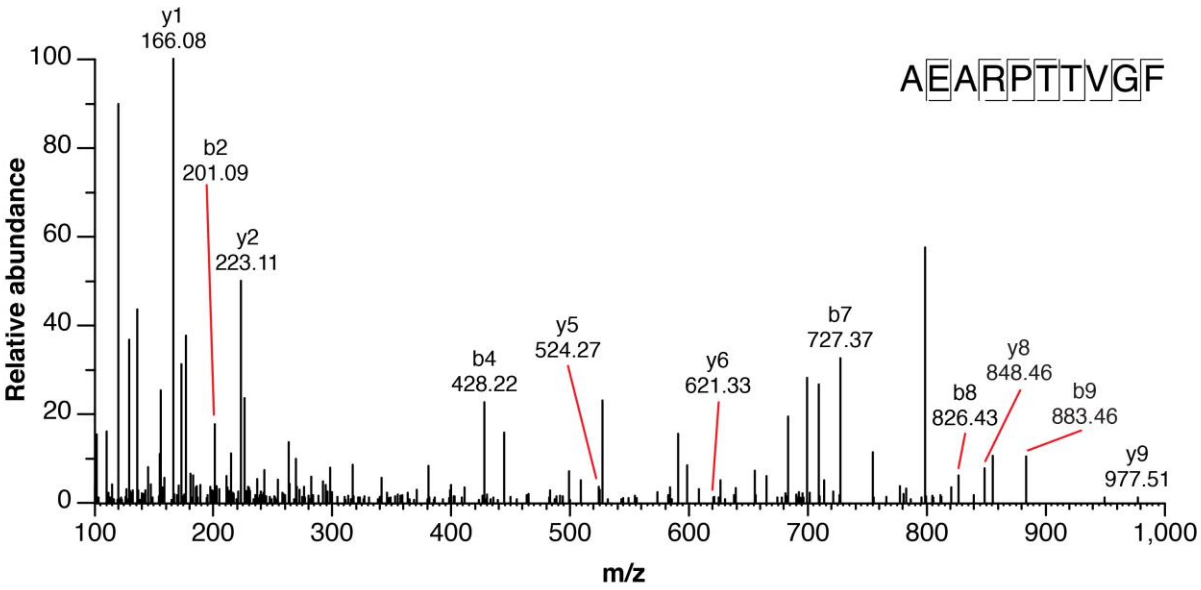

Synthetic peptide (related to Fig. 7)

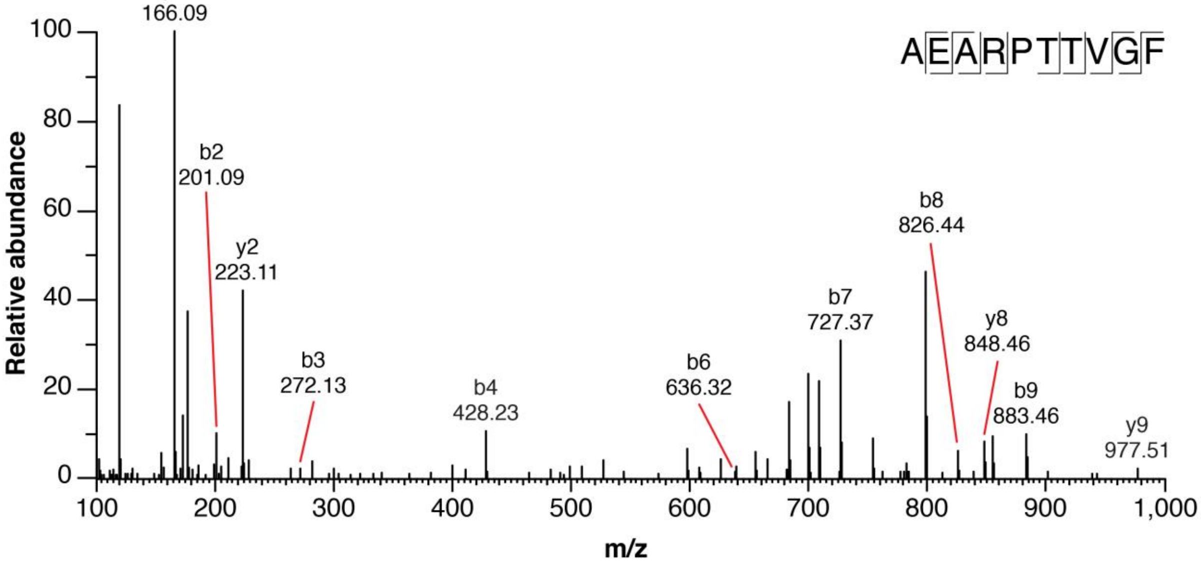

b

Endogenous peptide (related to Fig. 7)

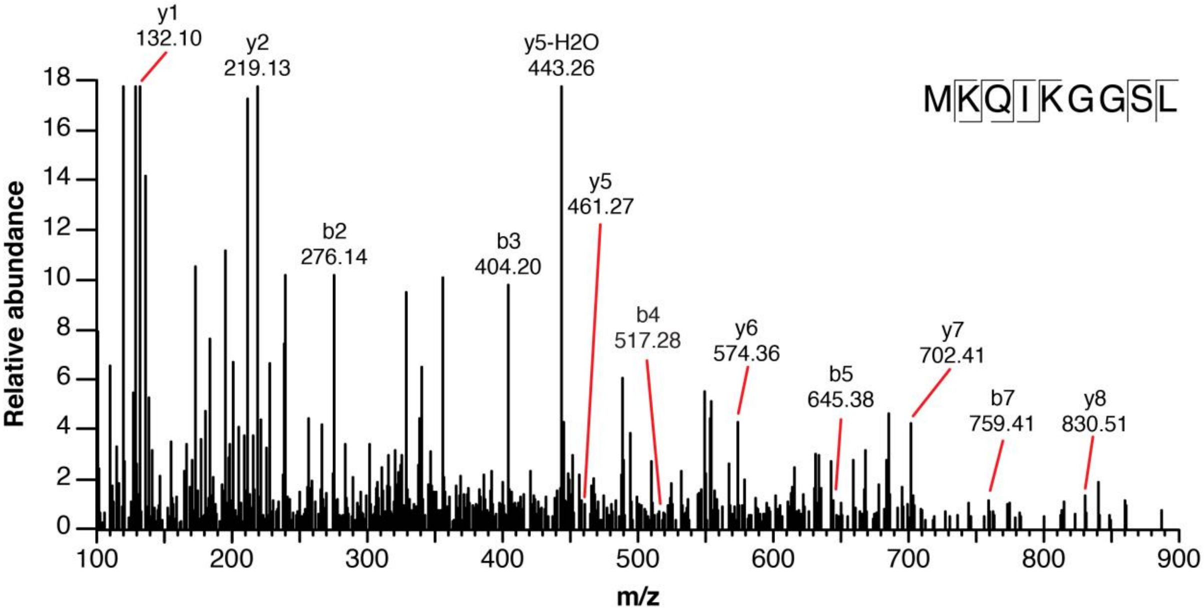

Synthetic peptide (related to Fig. 7)

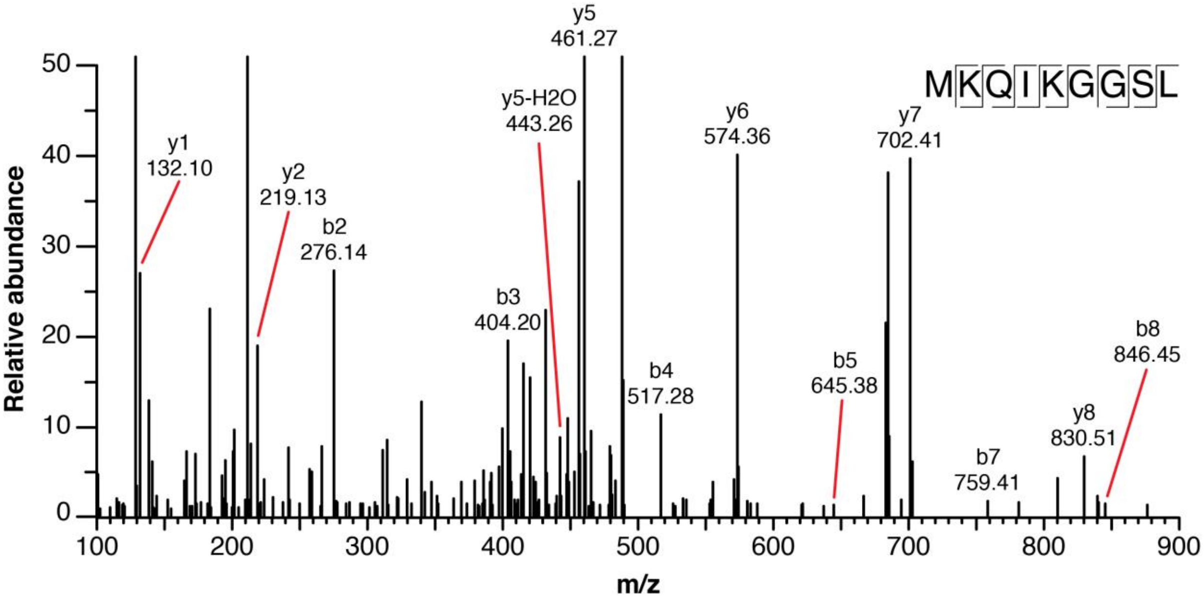

c

Endogenous peptide (related to Fig. 7)

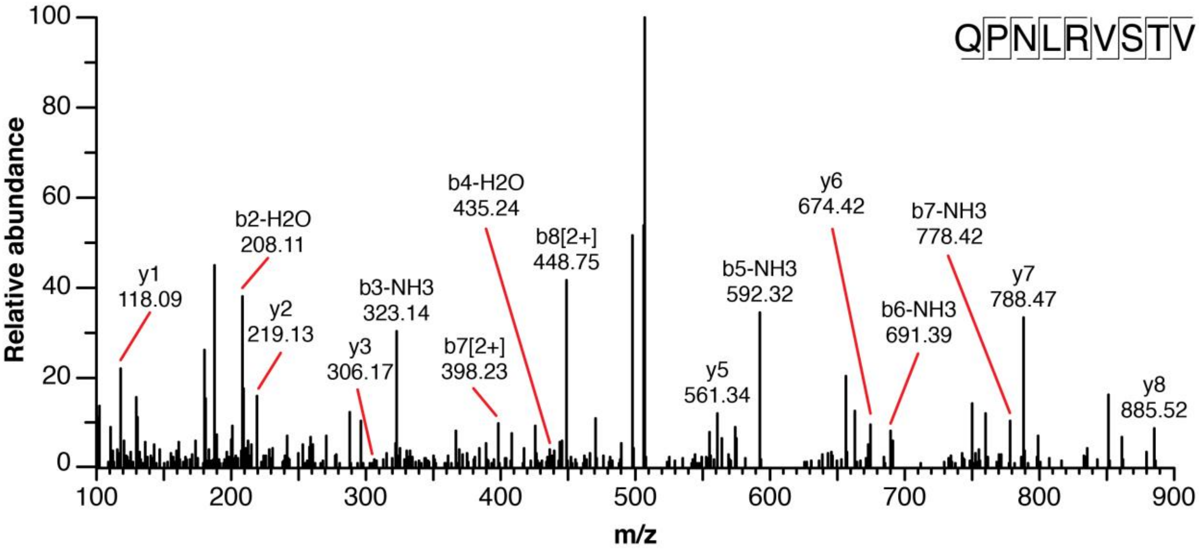

Synthetic peptide (related to Fig. 7)

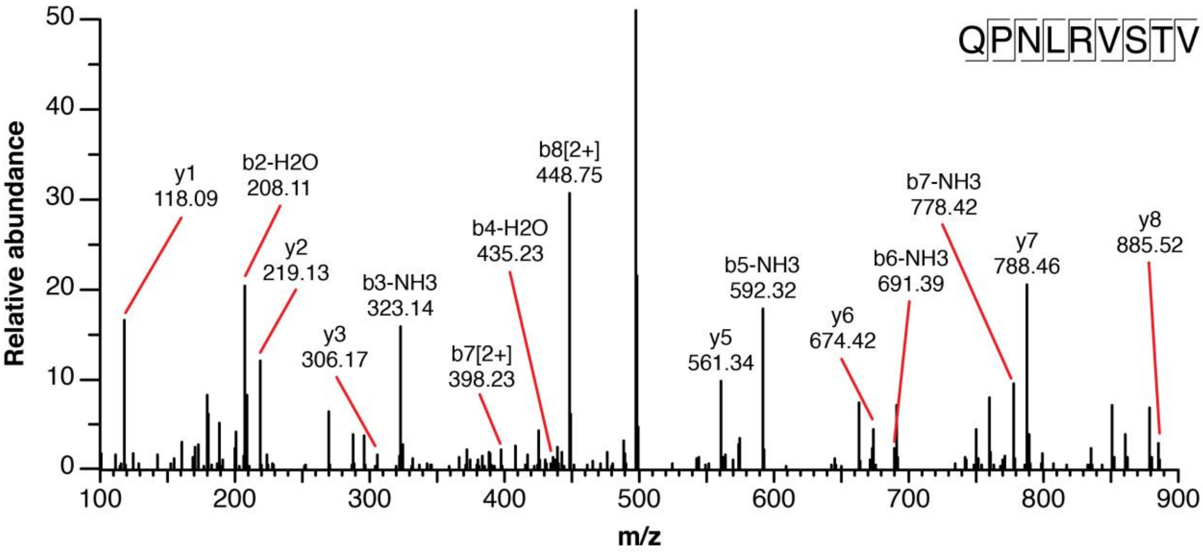

d

Endogenous peptide (related to Fig. 7)

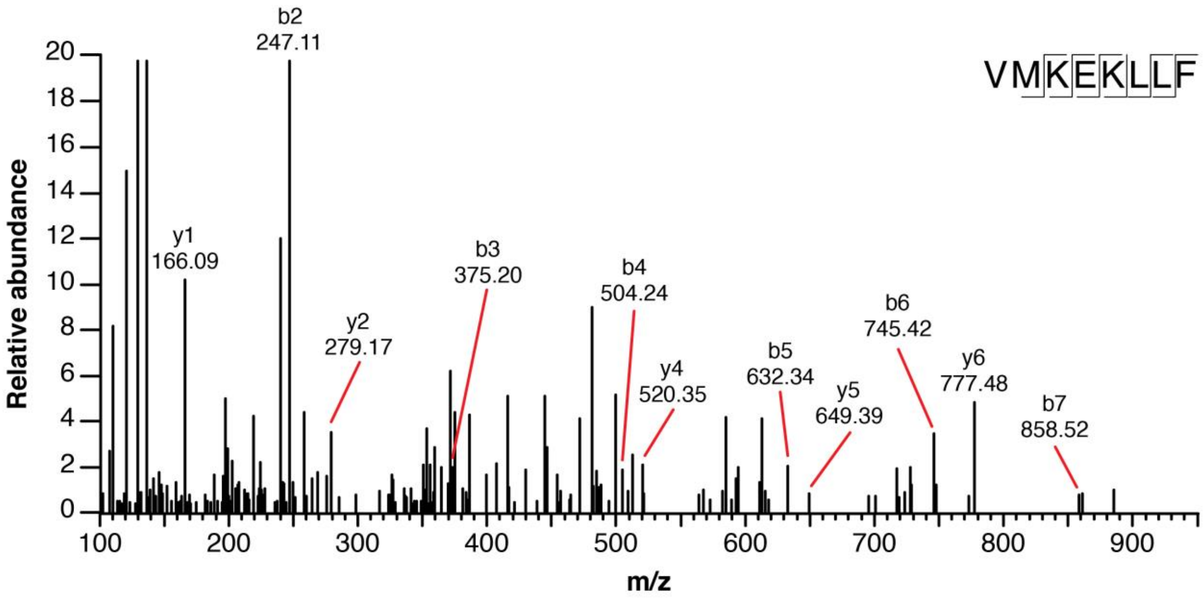

Synthetic peptide (related to Fig. 7)

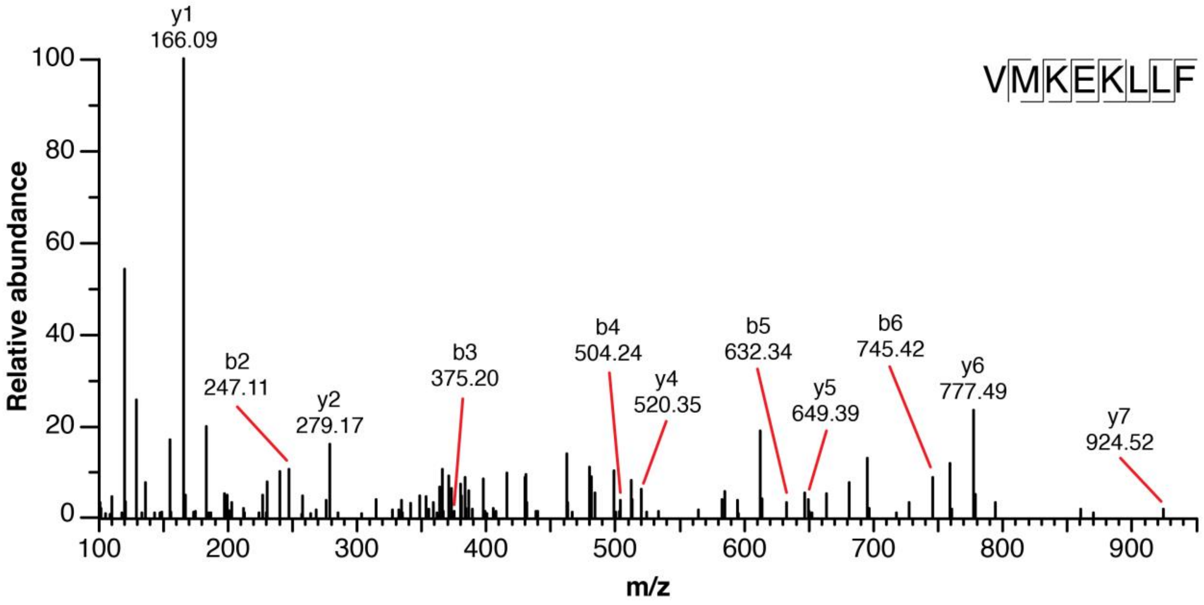

e

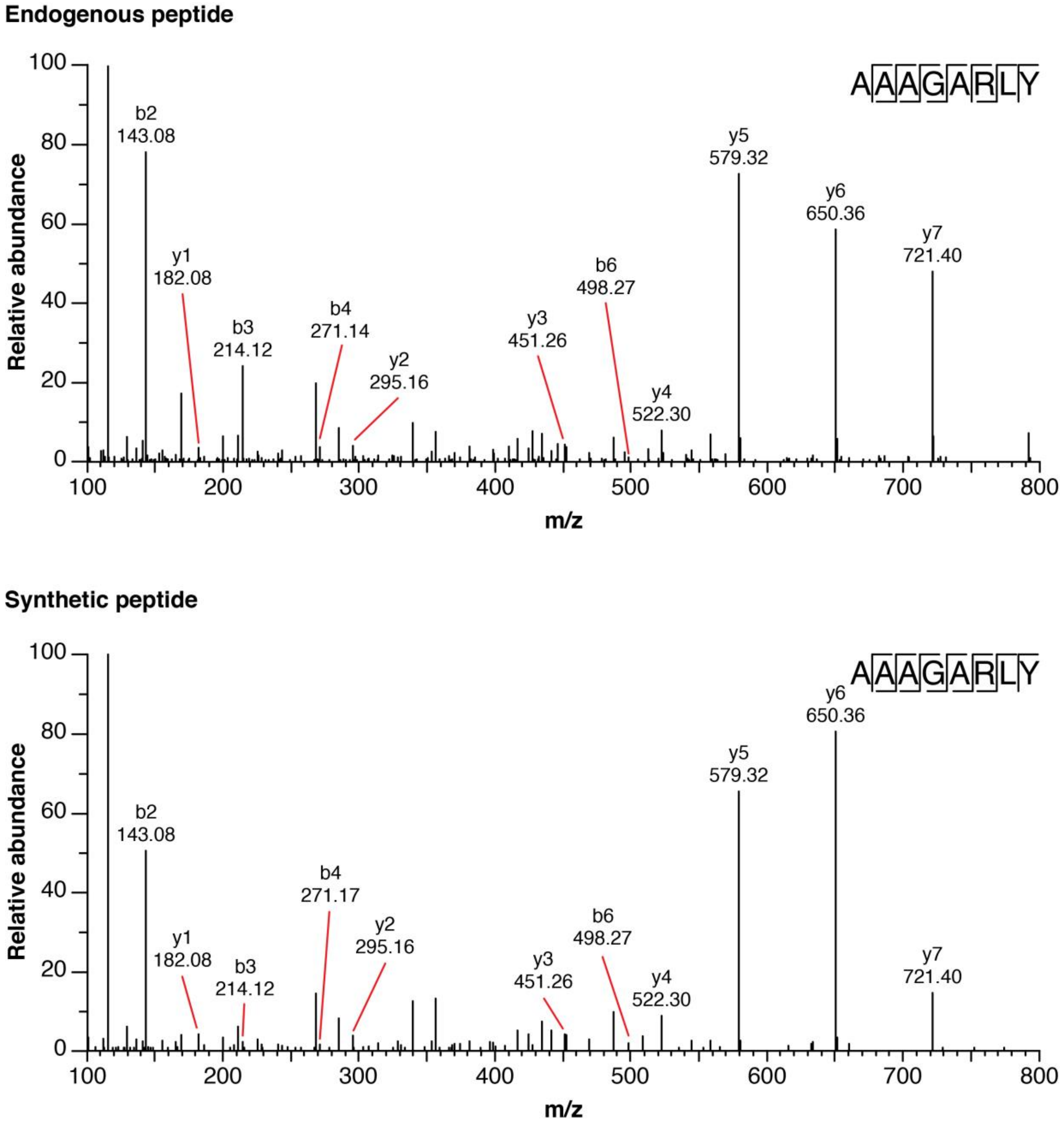

Endogenous peptide
AAAGARLY
Relative abundance
m/z
b2
143.08
y1
182.08
b3
214.12
b4
271.14
y2
295.16
y3
451.26
b6
498.27
y4
522.30
y5
579.32
y6
650.36
y7
721.40
Synthetic peptide
AAAGARLY
b2
143.08
y1
182.08
b3
214.12
b4
271.17
y2
295.16
y3
451.26
b6
498.27
y4
522.30
y5
579.32
y6
650.36
y7
721.40

f

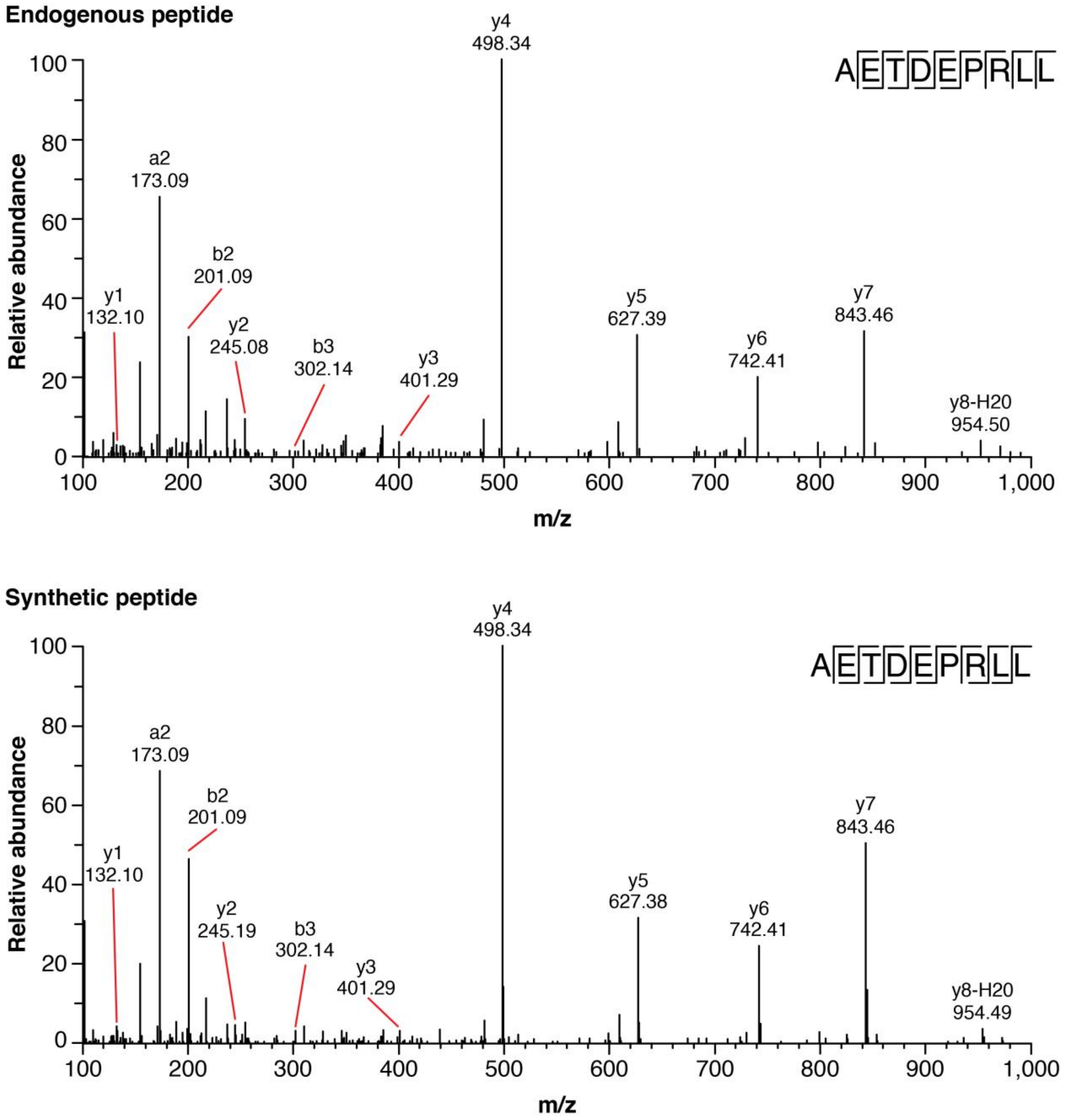
Endogenous peptide
AETDEPRLL
Relative abundance
m/z
y4
498.34
a2
173.09
b2
201.09
y1
132.10
y2
245.08
b3
302.14
y3
401.29
y5
627.39
y6
742.41
y7
843.46
y8-H20
954.50
Synthetic peptide
AETDEPRLL
Relative abundance
m/z
y4
498.34
a2
173.09
b2
201.09
y1
132.10
y2
245.19
b3
302.14
y3
401.29
y5
627.38
y6
742.41
y7
843.46
y8-H20
954.49

g

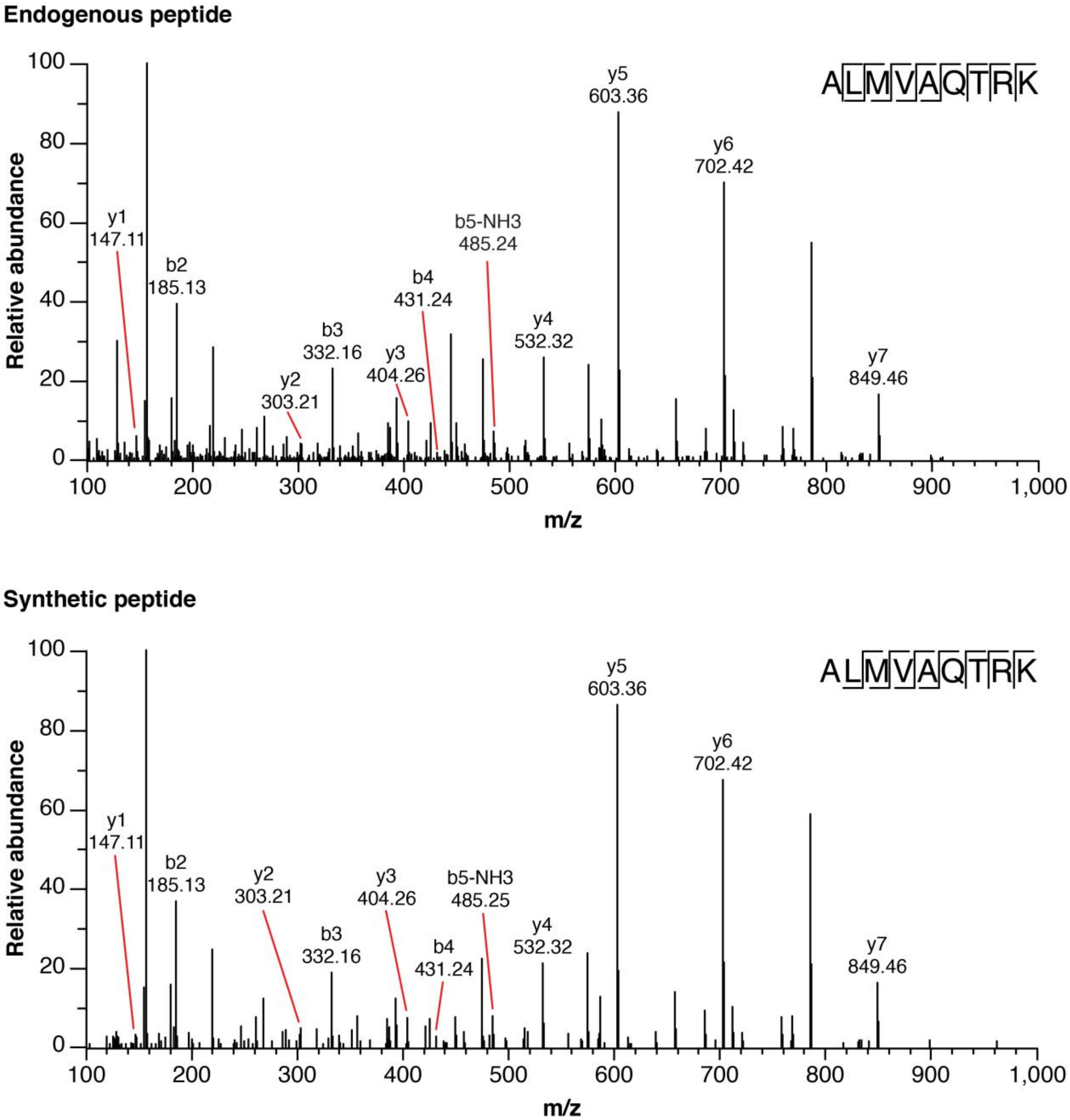
Endogenous peptide
ALMVAQTRK
Relative abundance
m/z
y1
147.11
b2
185.13
y2
303.21
b3
332.16
y3
404.26
b4
431.24
b5-NH3
485.24
y4
532.32
y5
603.36
y6
702.42
y7
849.46
Synthetic peptide
ALMVAQTRK
Relative abundance
m/z
y1
147.11
b2
185.13
y2
303.21
b3
332.16
y3
404.26
b4
431.24
b5-NH3
485.25
y4
532.32
y5
603.36
y6
702.42
y7
849.46

h

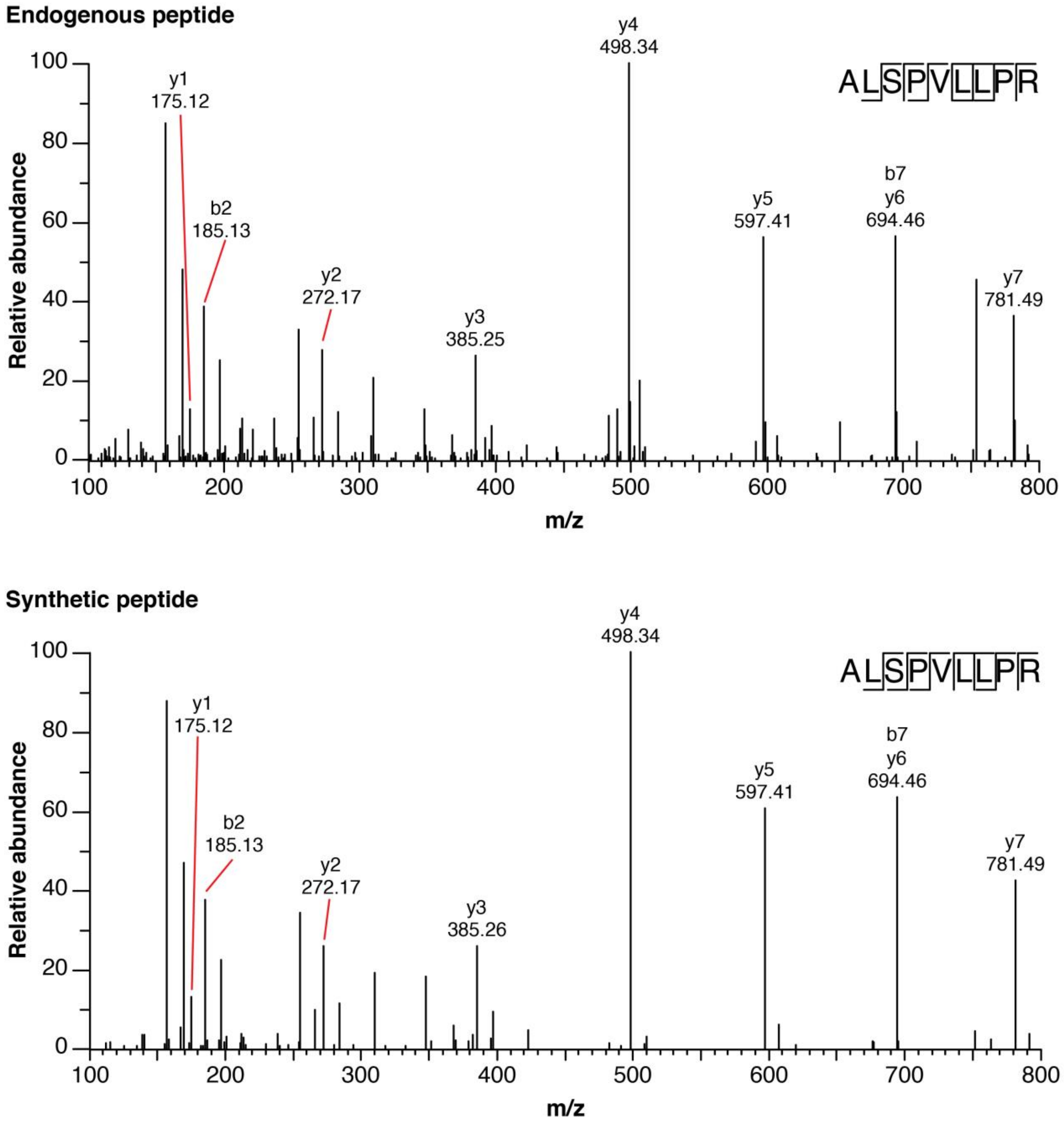
Endogenous peptide
ALSPVLLPR
Relative abundance
m/z
y1
175.12
b2
185.13
y2
272.17
y3
385.25
y4
498.34
y5
597.41
b7
y6
694.46
y7
781.49
0
20
40
60
80
100
100
200
300
400
500
600
700
800
Synthetic peptide
ALSPVLLPR
Relative abundance
m/z
y1
175.12
b2
185.13
y2
272.17
y3
385.26
y4
498.34
y5
597.41
b7
y6
694.46
y7
781.49
0
20
40
60
80
100
100
200
300
400
500
600
700
800

i

Endogenous peptide

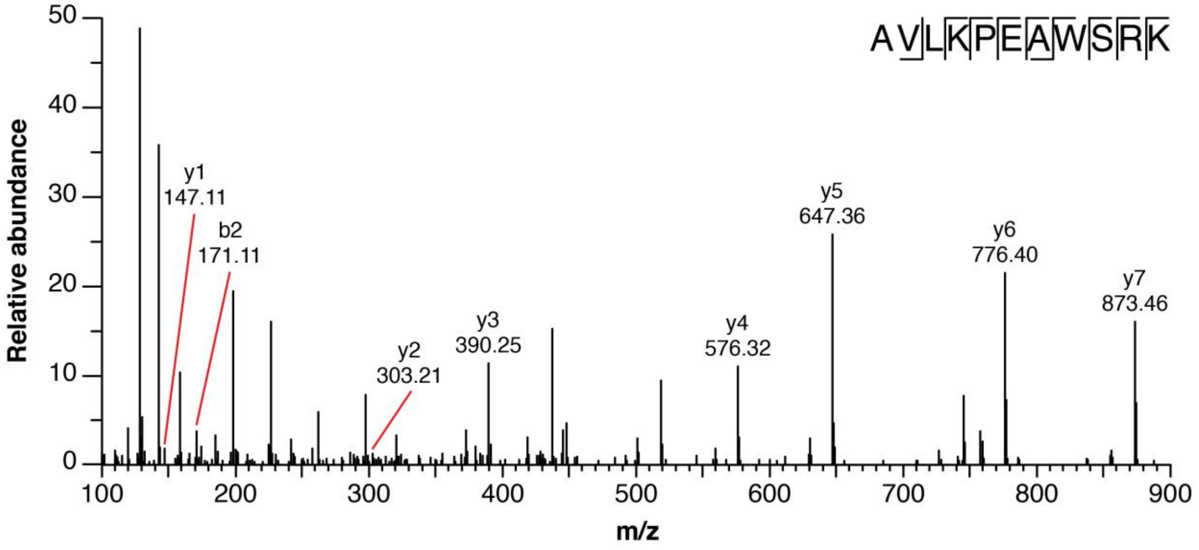

Synthetic peptide

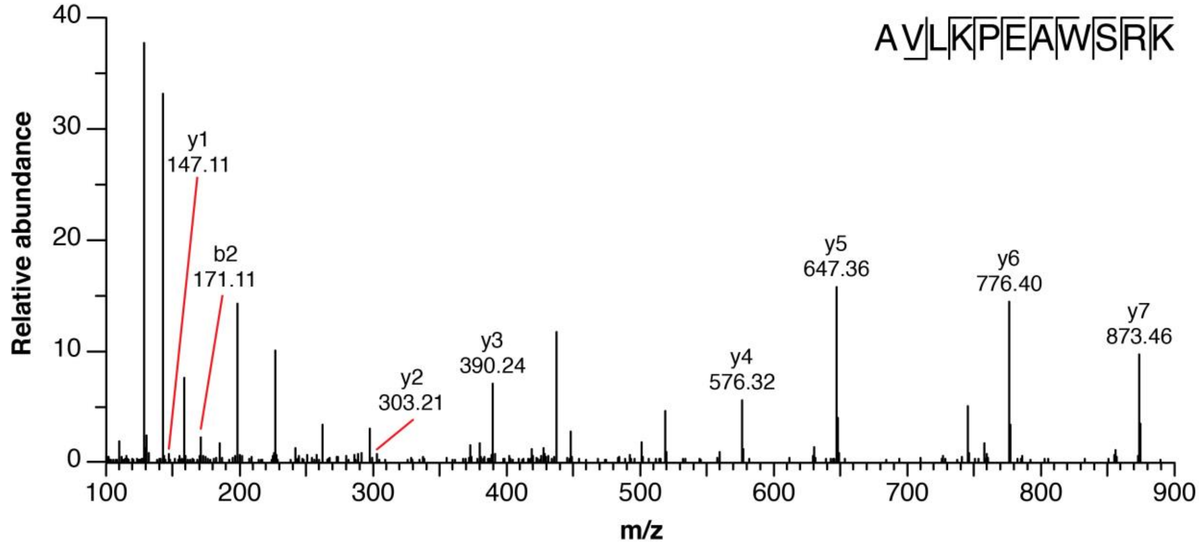

j

Endogenous peptide

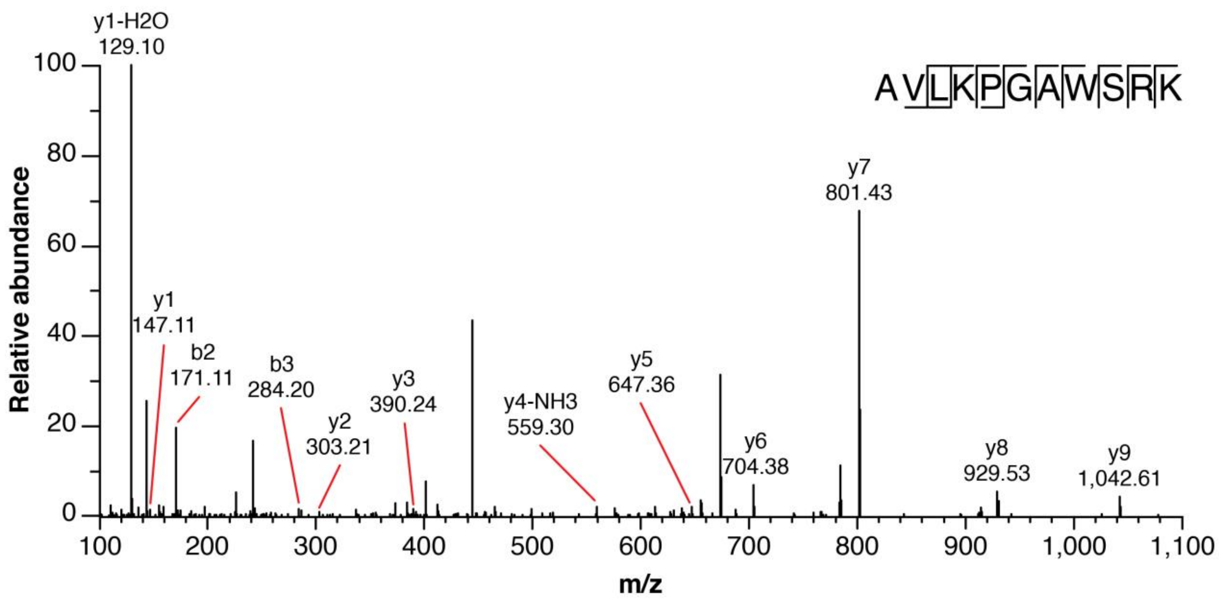

Synthetic peptide

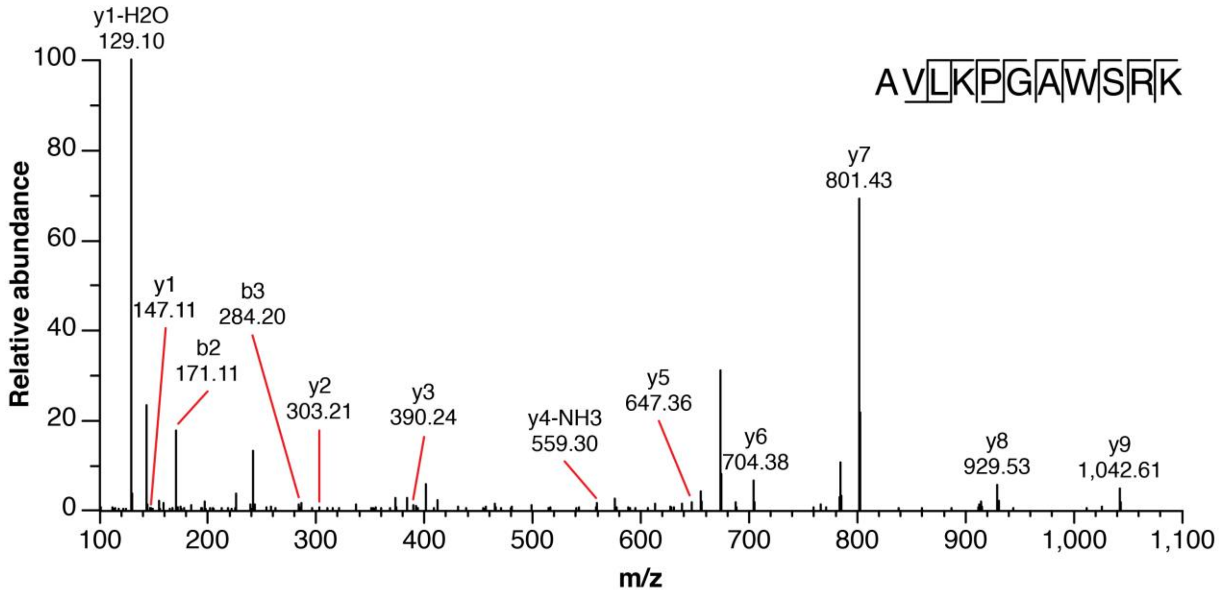

k

Endogenous peptide

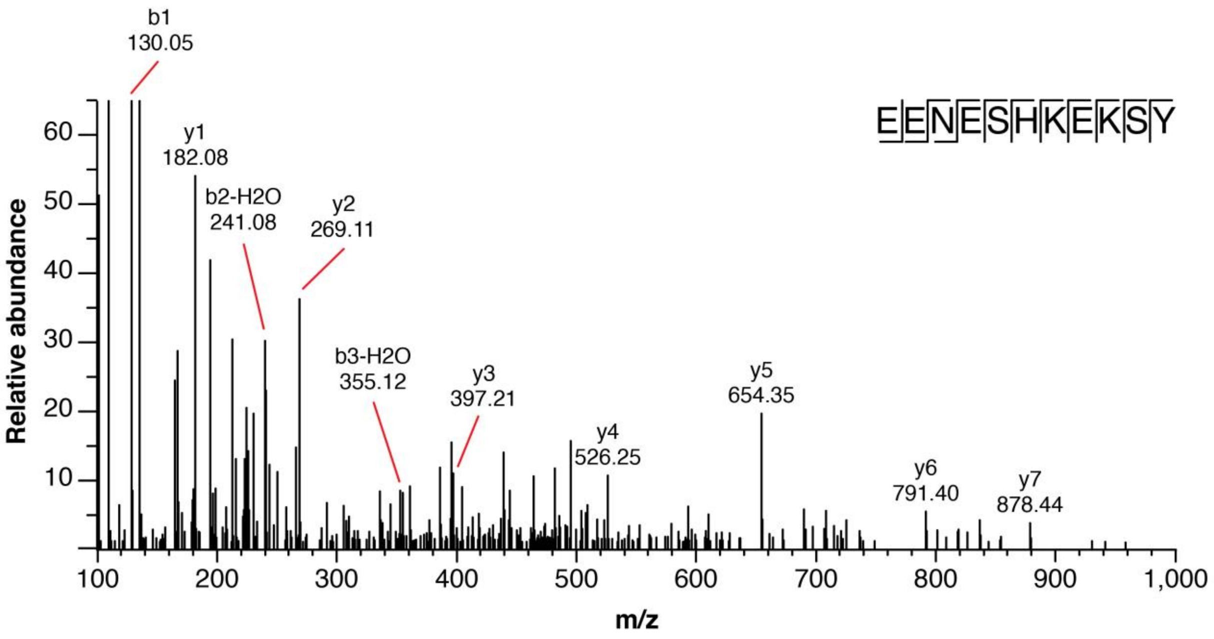

Synthetic peptide

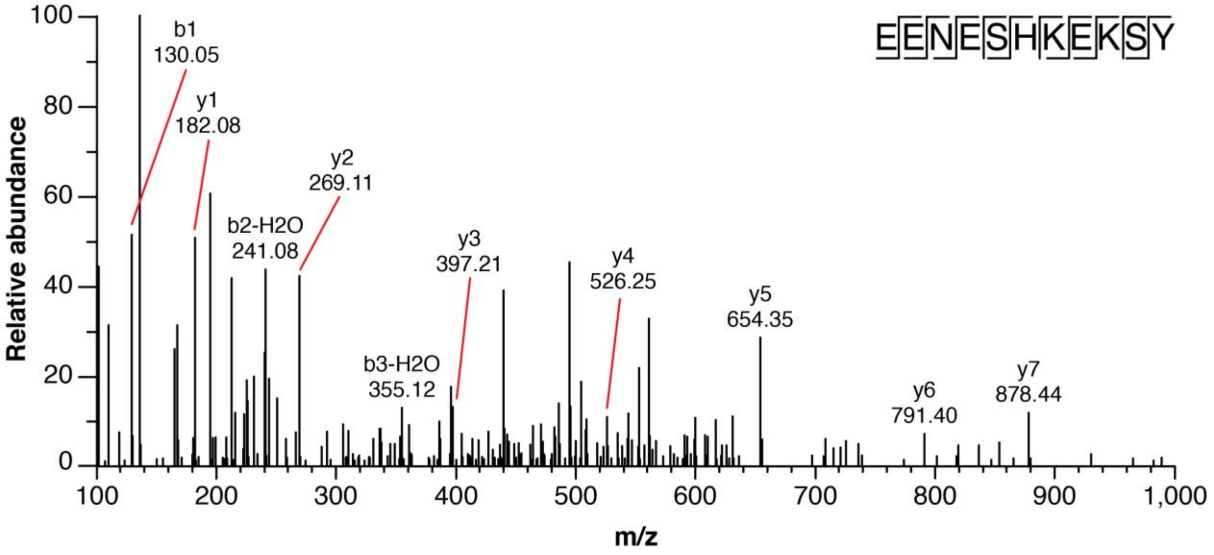

I

Endogenous peptide

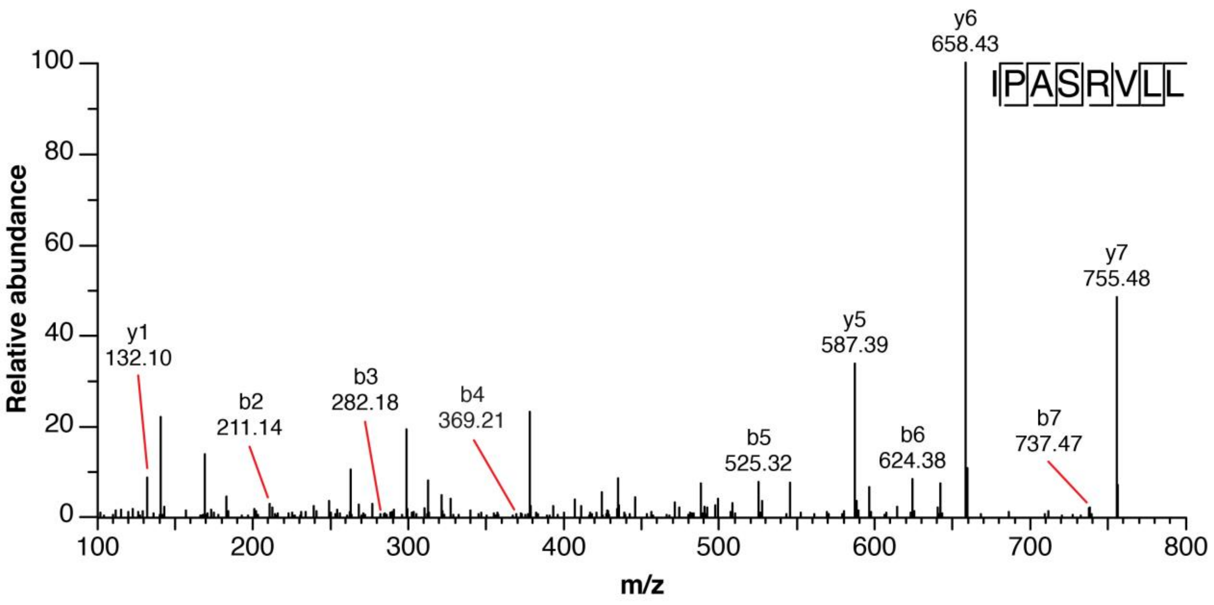

Synthetic peptide

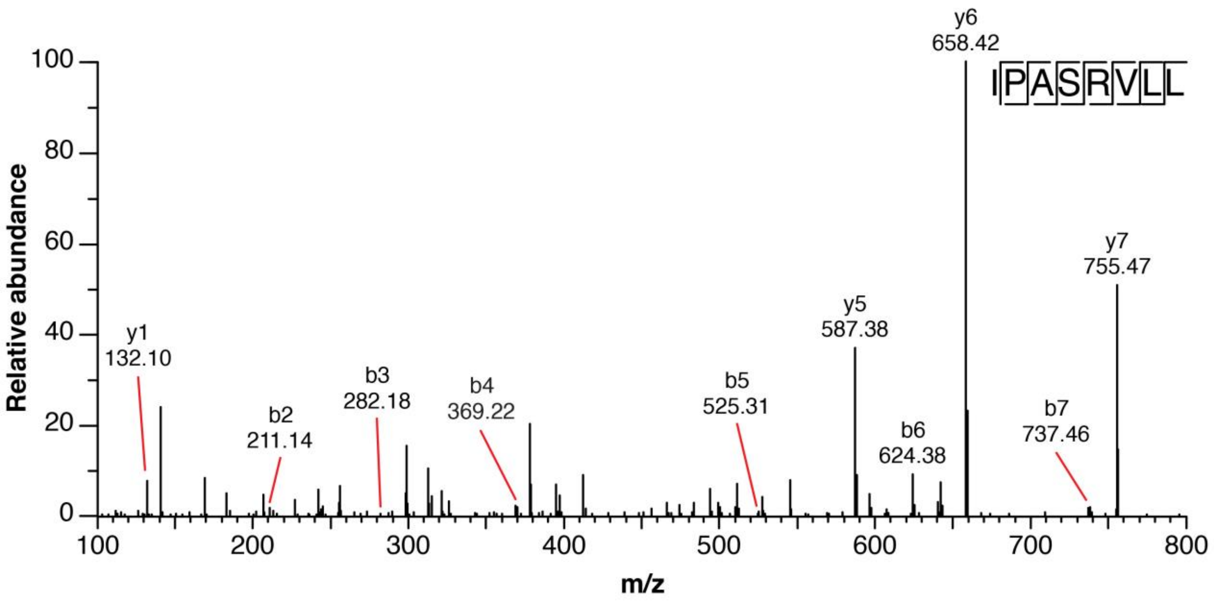

m

Endogenous peptide

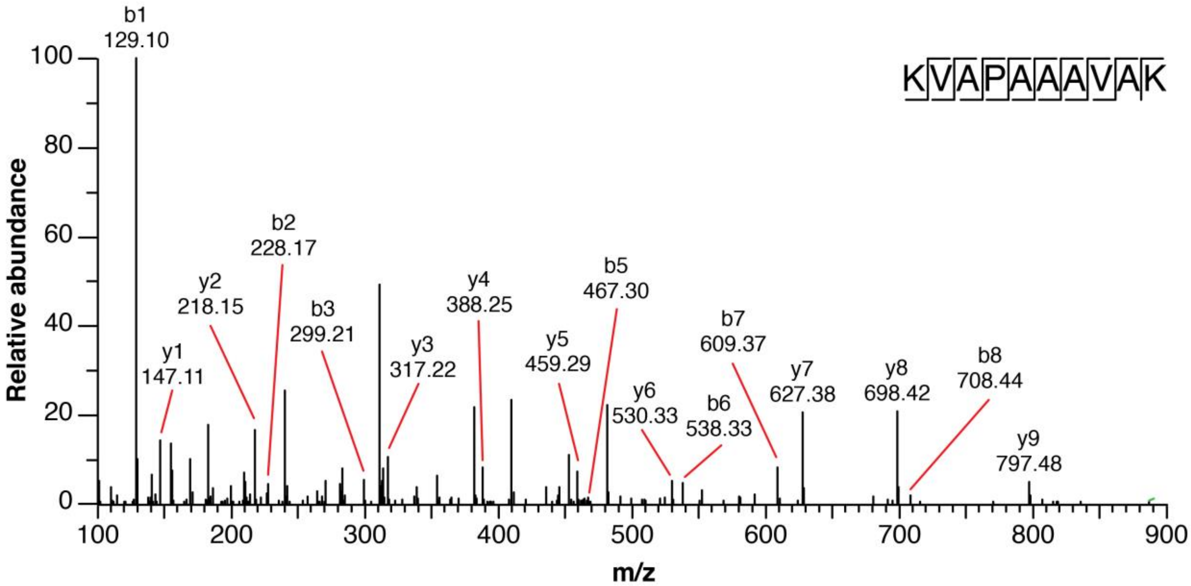

Synthetic peptide

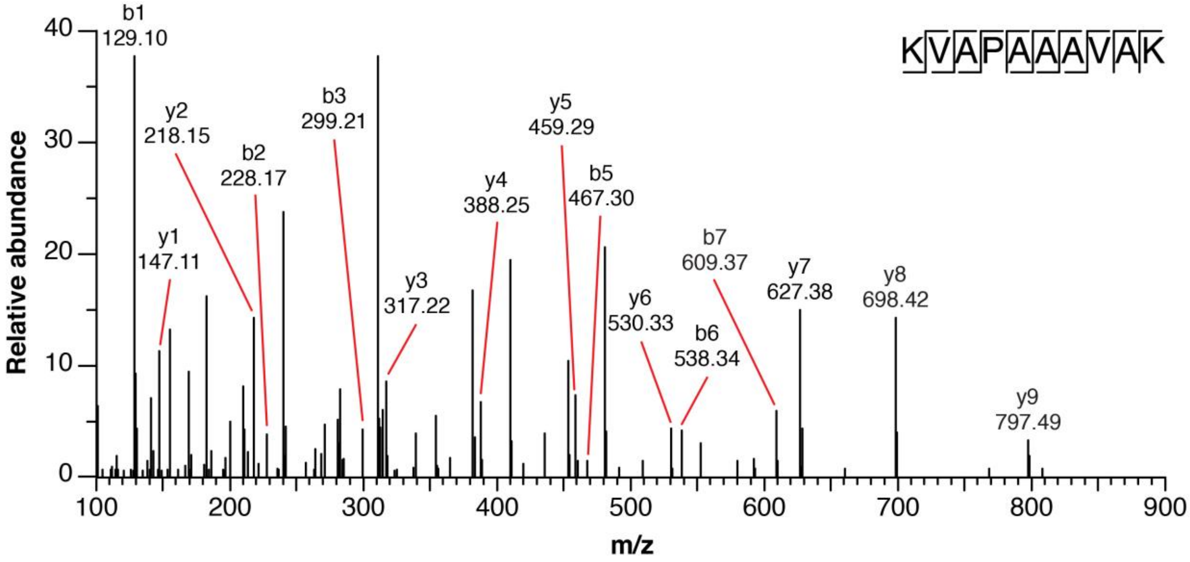

n

Endogenous peptide

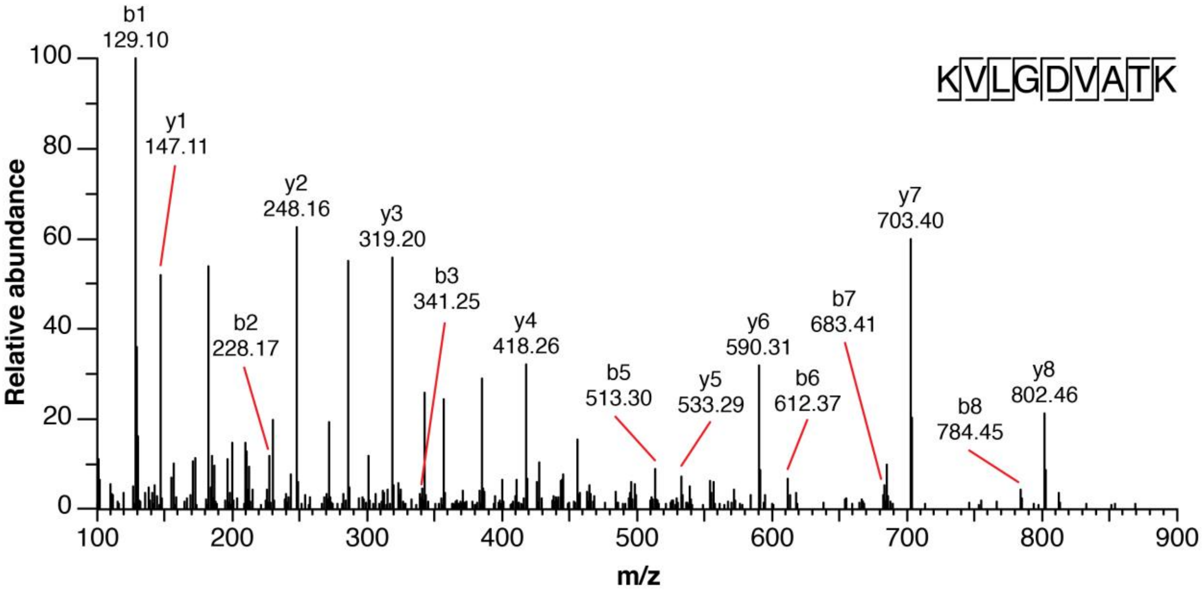

Synthetic peptide

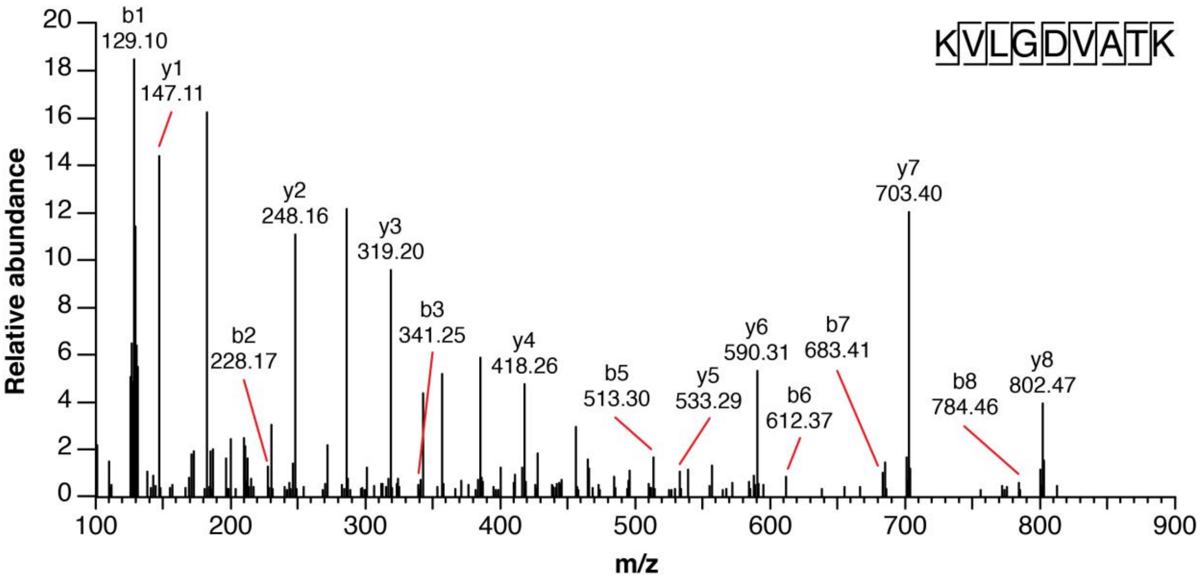

o

Endogenous peptide

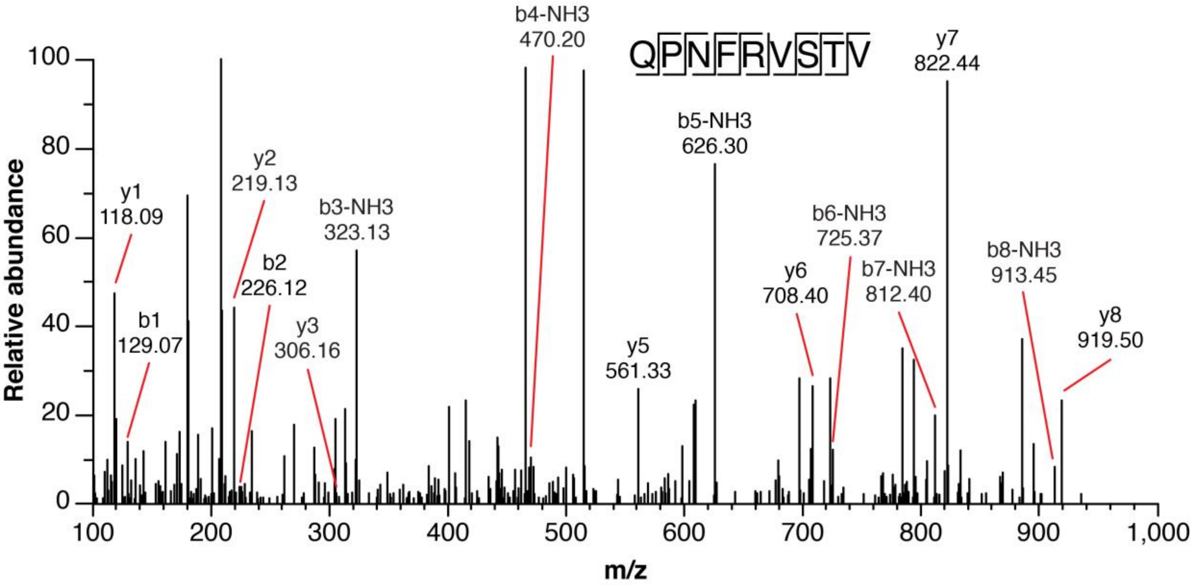

Synthetic peptide

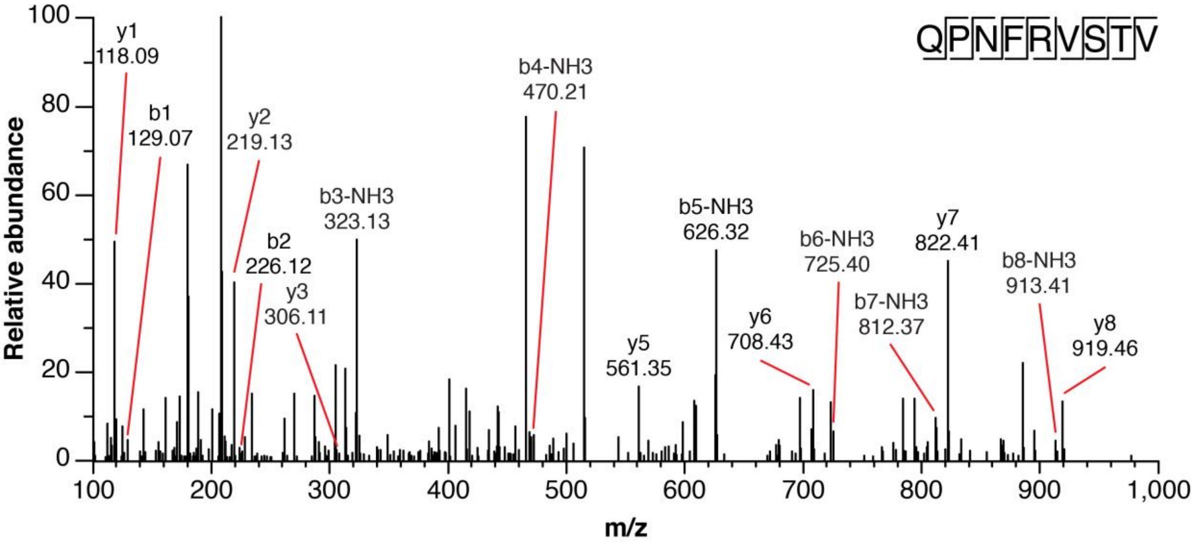

p

Endogenous peptide

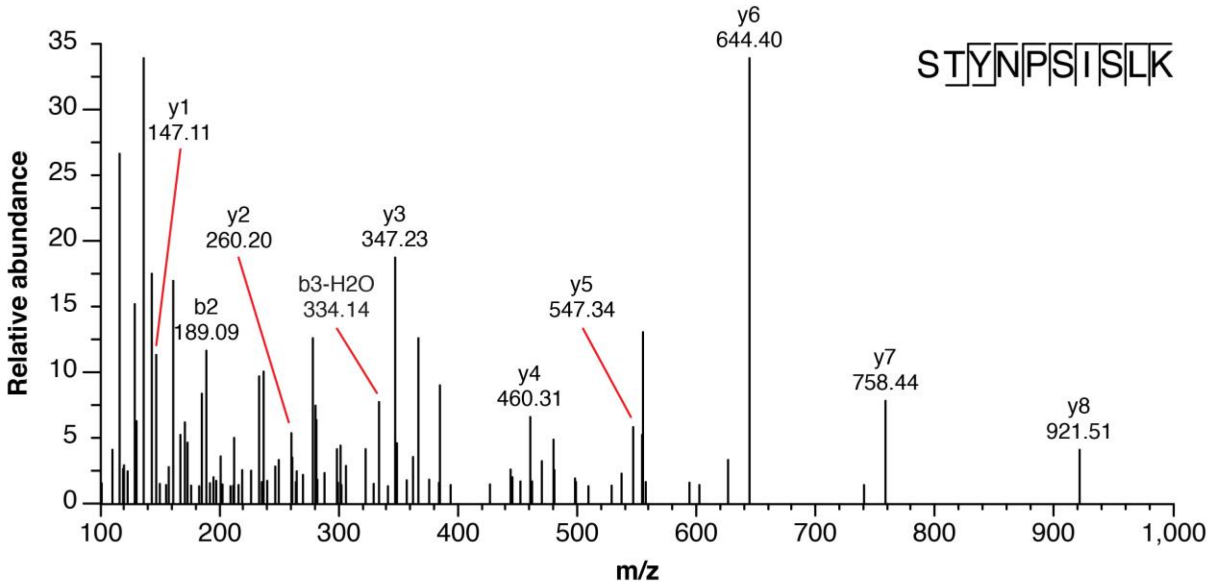

Synthetic peptide

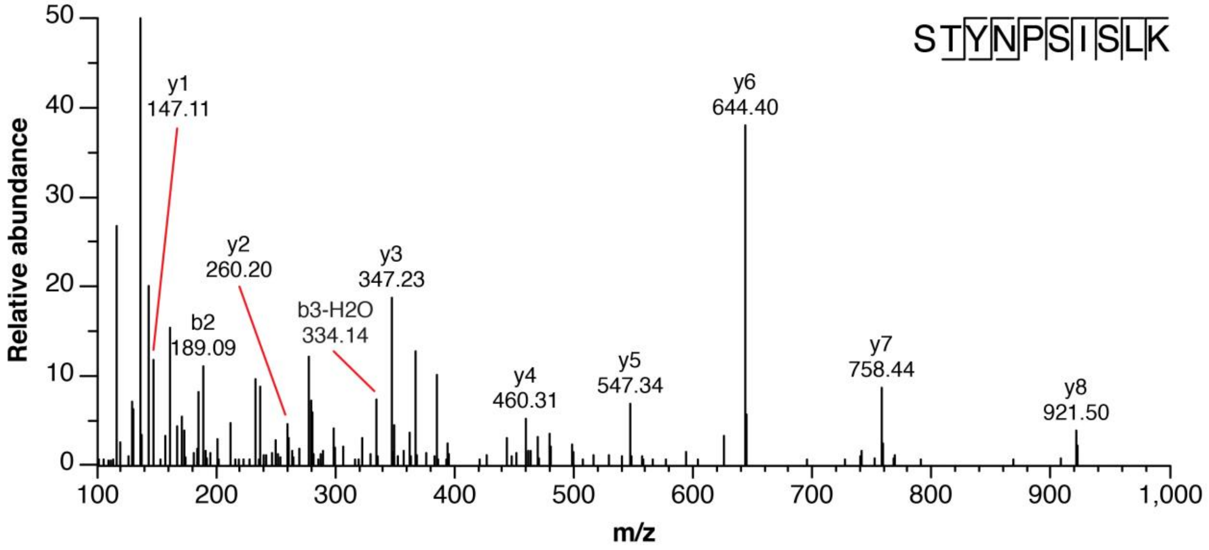

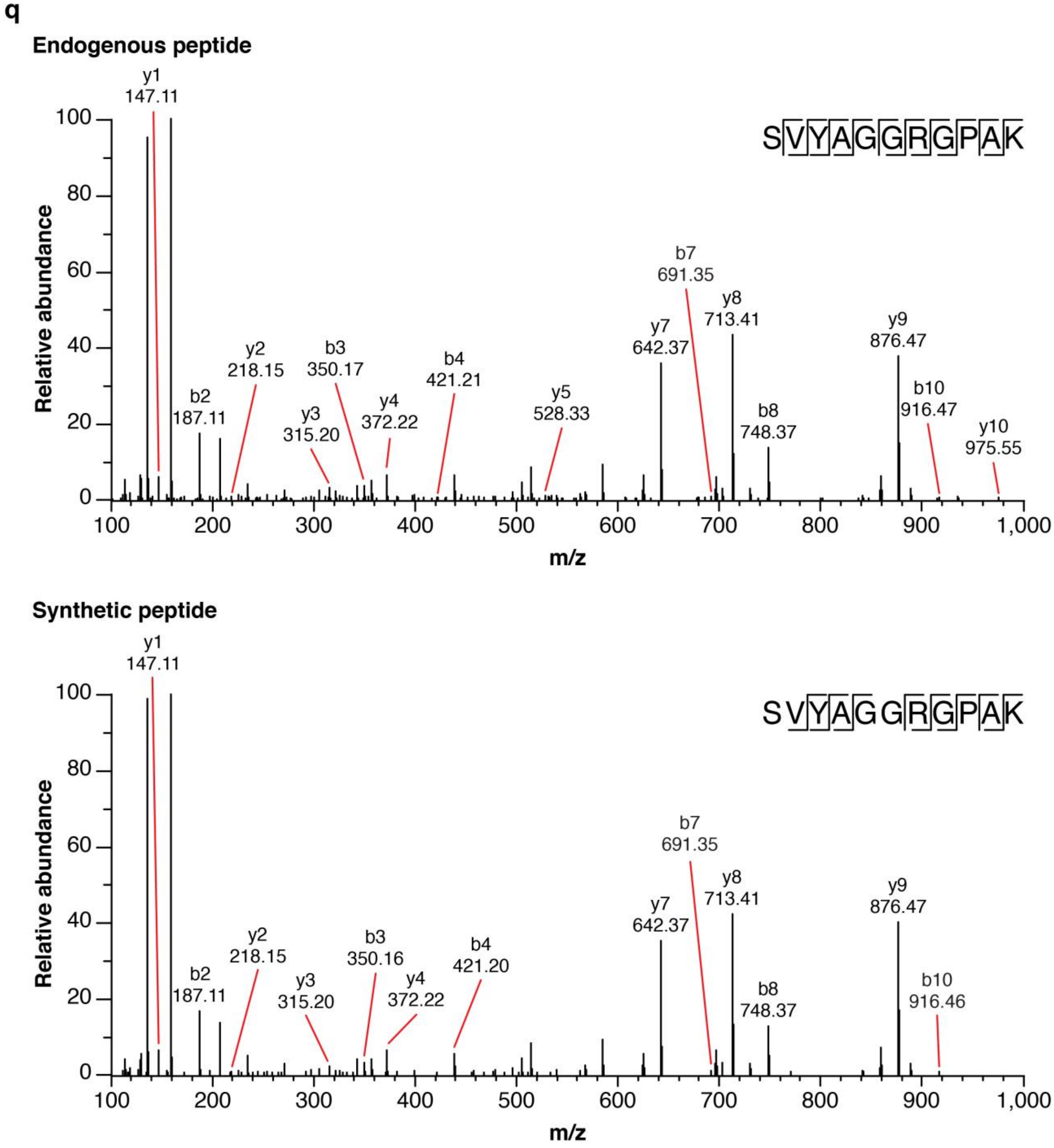
q
Endogenous peptide
y1
147.11
b2
187.11
y2
218.15
y3
315.20
b3
350.17
y4
372.22
b4
421.21
y5
528.33
y7
642.37
b7
691.35
y8
713.41
b8
748.37
y9
876.47
b10
916.47
y10
975.55
SVYAGGRGPAK
Relative abundance
m/z
Synthetic peptide
y1
147.11
b2
187.11
y2
218.15
y3
315.20
b3
350.16
y4
372.22
b4
421.20
y7
642.37
b7
691.35
y8
713.41
b8
748.37
y9
876.47
b10
916.46
SVYAGGRGPAK
Relative abundance
m/z

r

Endogenous peptide

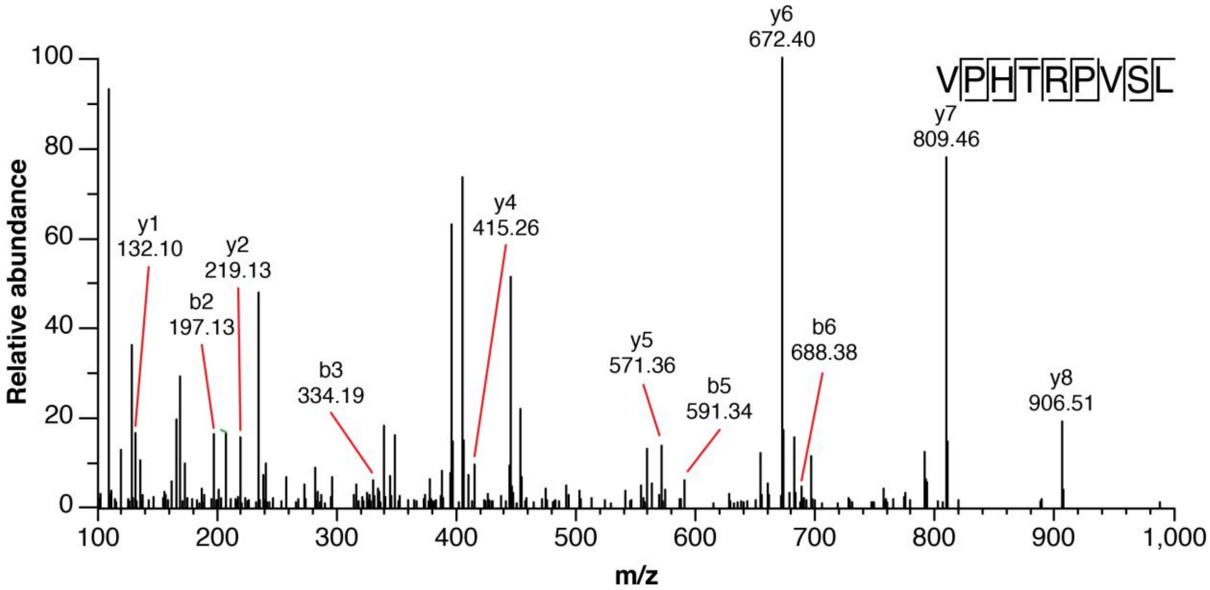

Synthetic peptide

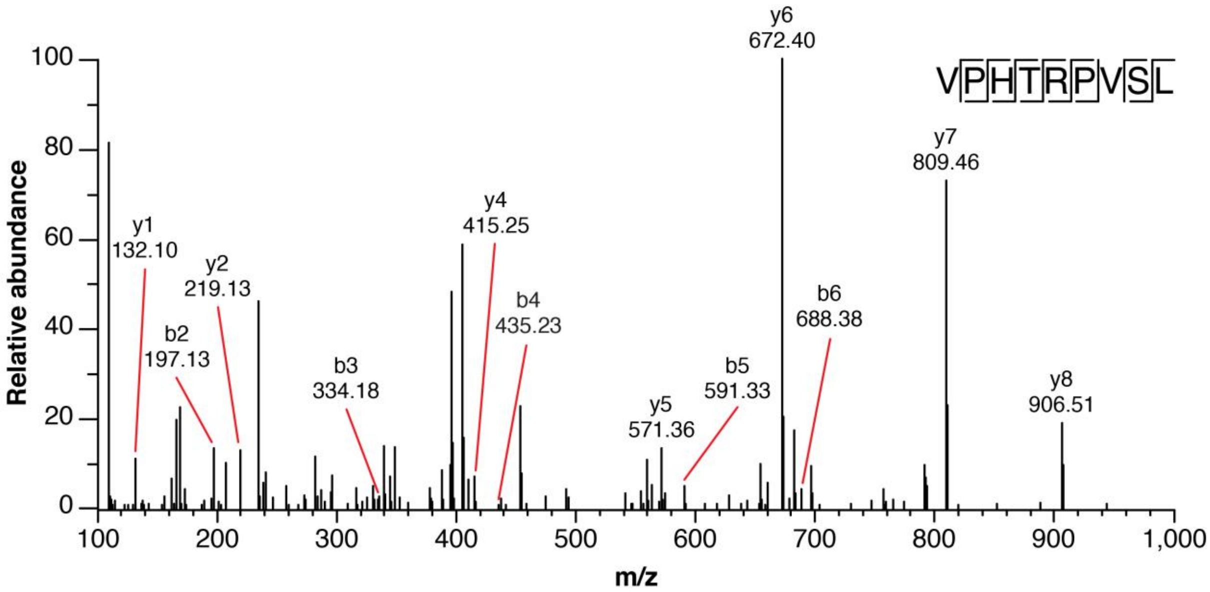

Supplementary Figure 2. MS validation of 18 cryptic MAPs. Among the 168 cryptic MAPs identified in our study, 18 were randomly selected and subjected to MS validation using synthetic version of them. (**a-d**) Four cryptic MAPs related to Fig. 7. (**e-r**) 14 other cryptic MAPs.

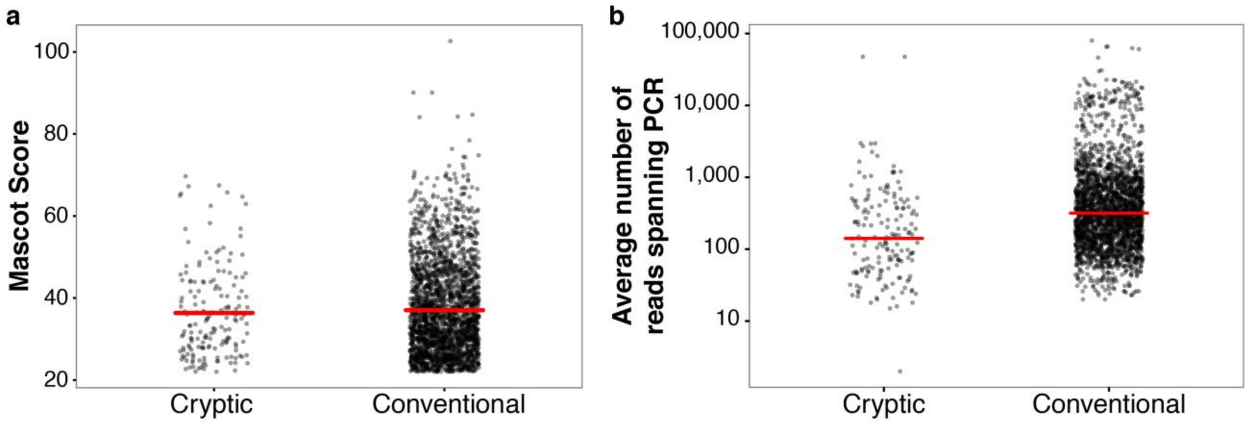

Supplementary Figure 3. Cryptic and conventional MAPs are similarly detected by mass spectrometry and RNA-seq. (**a**) Cryptic and conventional MAPs display similar Mascot score distributions. Dot plot representing the Mascot score distribution for cryptic and conventional MAPs, with the Mascot score being an indicator for the goodness of a peptide-spectrum match. (**b**) Cryptic and conventional MAPs derive from regions covered by our RNA-seq experiment. Dot plot showing the average number of reads spanning the peptide-coding regions (PCR) of cryptic and conventional MAPs obtained following TopHat mapping. In both panels, red lines depict the median of each group for the considered metrics.

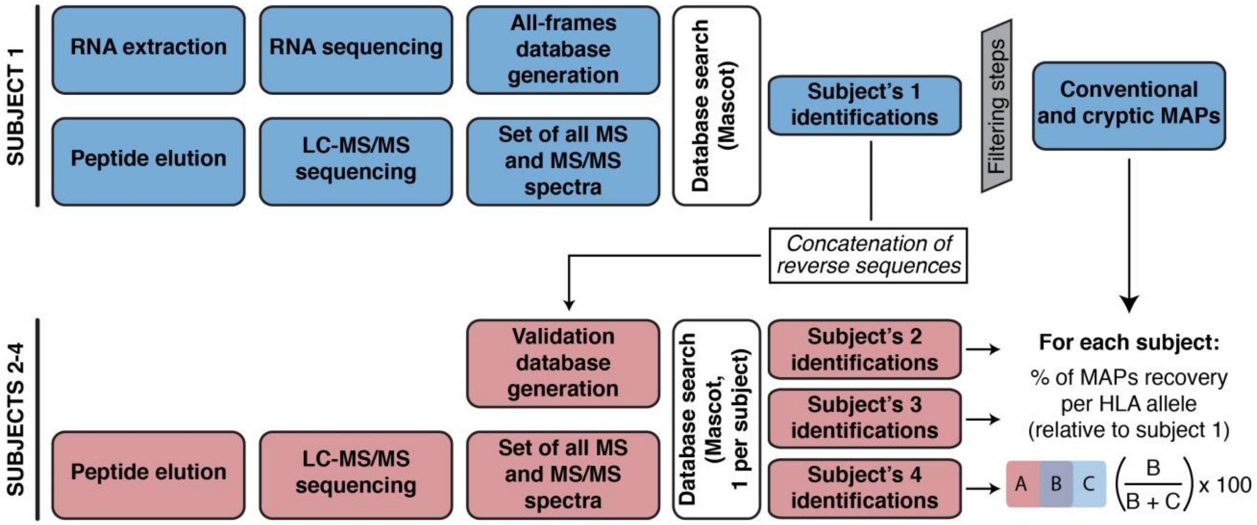

Supplementary Figure 4. Cryptic MAPs validation workflow. Detailed workflow of the analysis presented in Fig. 2a. We performed peptide elution on B-LCLs from three other subjects (subjects 2-4) that shared 4, 2 or no HLA allele with subject 1, respectively. Since we only wanted to validate MAPs from subject 1 rather than explore the whole immunopeptidome of subjects 2-4, we constructed a validation database that contained all identifications made in subject 1 concatenated with their reverse sequences. Identifications made in subjects 2-4 using Mascot were compared to the list of conventional and cryptic MAPs from subject 1 allowing us to compute a percentage of MAPs recovery per HLA allele relative to subject 1 for each subject.

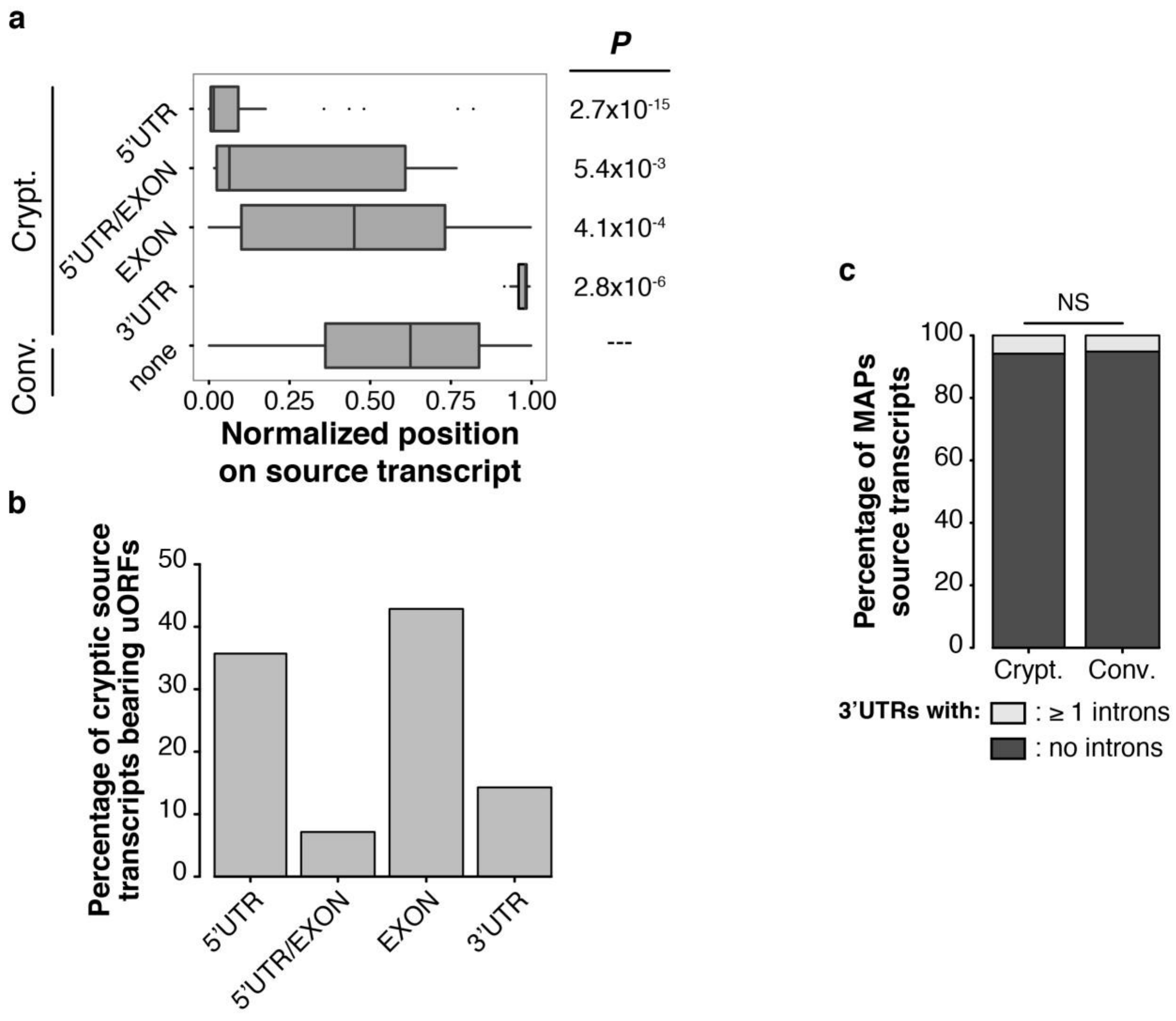

Supplementary Figure 5. Transcripts source of cryptic MAPs appear less stable than transcripts source of conventional MAPs. (**a**) Exonic cryptic PCRs are shifted towards the 5'end of source transcripts. Box plot depicting the normalized position in source transcript of PCRs for conventional and cryptic MAPs. For cryptic MAP PCRs, distinct box plots are depicted as a function of PCR genomic location. The two-sided Wilcoxon rank sum test was used to compare the location of conventional MAP PCRs to that of PCRs for various types of cryptic MAPs. Resulting *P*-values are indicated at the right of each box. (**b**) Transcripts containing uORFs do not solely generate 5'UTR and 5'UTR/EXON cryptic MAPs. Bar plot representing the percentage of each type of cryptic MAPs generated by transcripts bearing at least one uORF. (**c**) Transcripts source of conventional and cryptic MAPs contain the same proportion of 3'UTR intronic sequences. Bar plot depicting the percentage of transcripts containing no (dark gray) or at least one (light gray) intronic sequence in their 3'UTR for both conventional and cryptic MAPs. Statistical significance was assessed using a two-sided Fisher's exact test (NS: not significant, $P = 6.53x10^{-1}$).

SUPPLEMENTARY TABLES

Supplementary Table 1. HLA allotypes presented by subject 1-4

	A*01:01	A*02:01	A*03:01	A*29:02	B*08:01	B*18:01	B*39:24	B*44:03	B*57:01	Number of shared HLA with Subject 1
**Subject 1**			x	x	x			x		
**Subject 2**			x	x	x			x		4
**Subject 3**		x		x				x	x	2
**Subject 4**	x	x				x	x			0

Supplementary Table 2. Rare codon usage in conventional vs. cryptic MAP source transcripts or ORFs

	Conventional MAP source transcripts	Cryptic MAP source ORFs
Rare codons	464,739	3,030
Common codons	767,785	5,119

Supplementary Table 3. Rare codon usage in MAP vs. non-MAP source transcripts or ORFs

	MAP source transcripts or ORFs	Non-MAP source transcripts
Rare codons	467,769	12,118,695
Common codons	772,904	22,909,893
